# The Optimal Dosage and Duration of ω-3 PUFA Supplementation in Heart Failure Management: Evidence from a Network Meta-Analysis

**DOI:** 10.1016/j.advnut.2025.100366

**Published:** 2025-01-11

**Authors:** Ping-Tao Tseng, Bing-Yan Zeng, Chih-Wei Hsu, Chih-Sung Liang, Brendon Stubbs, Yen-Wen Chen, Tien-Yu Chen, Wei-Te Lei, Jiann-Jy Chen, Yow-Ling Shiue, Kuan-Pin Su

**Affiliations:** 1 Institute of Precision Medicine, National Sun Yat-sen University, Kaohsiung City, Taiwan; 2 Institute of Biomedical Sciences, National Sun Yat-sen University, Kaohsiung, Taiwan; 3 Department of Psychology, College of Medical and Health Science, Asia University, Taichung, Taiwan; 4 Prospect Clinic for Otorhinolaryngology and Neurology, Kaohsiung, Taiwan; 5 Department of Internal Medicine, E-Da Dachang Hospital, I-Shou University, Kaohsiung, Taiwan; 6 Department of Psychiatry, Kaohsiung Chang Gung Memorial Hospital and Chang Gung University College of Medicine, Kaohsiung, Taiwan; 7 Department of Psychiatry, Beitou Branch, Tri-Service General Hospital, Taipei, Taiwan; 8 School of Medicine, National Defense Medical Center, Taipei, Taiwan; 9 Department of Psychiatry, National Defense Medical Center, Taipei, Taiwan; 10 Department of Psychological Medicine, Institute of Psychiatry, Psychology and Neuroscience, King’s College London, London, United Kingdom; 11 Department of Sport Science, University of Vienna, Wien, Austria; 12 Department of Psychiatry, Tri-Service General Hospital, Taipei, Taiwan; 13 Institute of Brain Science, National Yang Ming Chiao Tung University, Taipei, Taiwan; 14 Section of Immunology, Rheumatology, and Allergy Department of Pediatrics, Hsinchu Mackay Memorial Hospital, Hsinchu City, Taiwan; 15 Graduate Institute of Clinical Medical Sciences, College of Medicine, Chang Gung University, Taoyuan City, Taiwan; 16 Department of Otorhinolaryngology, E-Da Cancer Hospital, I-Shou University, Kaohsiung, Taiwan; 17 Mind-Body Interface Research Center (MBI-Lab), China Medical University Hospital, Taichung, Taiwan; 18 College of Medicine, China Medical University, Taichung, Taiwan; 19 An-Nan Hospital, China Medical University, Tainan, Taiwan

**Keywords:** network meta-analysis, ω-3 polyunsaturated fatty acid, PUFA, EPA, DHA, heart failure, LVEF, cardiovascular disease

## Abstract

Heart failure is a progressive condition associated with a high mortality rate. Despite advancements in treatment, many patients continue to experience less-than-ideal outcomes. ω-3 (n–3) polyunsaturated fatty acids (PUFAs) have been studied as a potential supplementary therapy for heart failure, but the optimal dosage and duration of supplementation remain unclear. This network meta-analysis (NMA) aimed to assess the efficacy of various n–3 PUFA supplementation regimens in patients with heart failure, focusing on dose-dependent and time-dependent effects. We conducted a systematic search for randomized controlled trials (RCTs) on n–3 PUFA supplementation in heart failure till 13 September, 2024. The primary outcome was the change in heart function, specifically left ventricular ejection fraction. Secondary outcomes included changes in peak oxygen consumption (VO_2_), blood B-type natriuretic peptide concentrations, and quality of life. The safety analysis focused on dropout rates (i.e., patients leaving the study for any reason before completion) and all-cause mortality. A frequentist-based NMA was performed. This NMA, which included 14 RCTs with 9075 participants (mean age, 66.0 y; 23.3% female), found that high-dose n–3 PUFA supplementation (2000–4000 mg/d) over a duration of ≥1 y significantly improved left ventricular ejection fraction and peak VO_2_ compared with those of control groups. Lower doses and shorter treatment periods did not produce the same benefits. No significant differences were found in dropout rates or all-cause mortality between the n–3 PUFAs and control groups. Long-term, high-dose n–3 PUFA supplementation, particularly with a predominance of docosahexaenoic acid or eicosapentaenoic acid, enhances cardiac function in patients with heart failure without increasing risk of adverse events. Further well-designed RCTs with long treatment durations (i.e., >1 y) and stringent heart failure inclusion criteria are necessary to confirm these findings and reduce potential biases.

This trial was registered at PROSPERO as CRD42024590476.

**Statement of Significance**

Long-term, high-dose n–3 PUFA supplementation significantly enhances heart failure outcomes, particularly in improving left ventricular ejection fraction and peak VO_2_, without raising dropout rates or all-cause mortality. These findings advocate for the inclusion of specific n–3 PUFAs regimens in the management of heart failure.

## Introduction

Heart failure represents the final stage of numerous cardiovascular diseases, driven by complex underlying mechanisms [1], including neuroendocrine activation, the release of proinflammatory cytokines, platelet activation, and endothelial dysfunction [2]. As heart failure progresses, pronounced inflammatory responses, such as the overproduction of TNF-α and IL-6, result in damage to the vascular walls and reduced myocardial efficiency [3]. Despite advancements in pharmacologic therapies, the outlook for patients with heart failure remains poor, with only a 50% of 5-y survival rate [4], and ∼75% of patients succumbing within 10 y of diagnosis [5]. In addition to its high mortality rate, heart failure is often associated with diminished physical capacity and quality of life [1], along with burdensome symptoms such as dyspnea, disability, impaired daily functioning, frequent hospitalizations, and ultimately, death [6]. Several innovative nonpharmacologic treatments, including nutritional supplements, have garnered attention for their potential to address the multiple pathophysiologic processes in heart failure [7].

ω-3 (n–3) PUFAs are among the most extensively studied nutritional interventions. These fatty acids have demonstrated a range of benefits in heart failure management, such as reducing inflammation markers [8,9]. The GISSI-HF trial revealed that n–3 PUFA supplementation significantly improved left ventricular ejection fraction (LVEF), a crucial marker of cardiac function and remodeling [10]. Additional studies have shown improvements in B-type natriuretic peptide (BNP) concentrations [11], decreased adverse ventricular remodeling [12], enhanced peak oxygen consumption (peak VO_2_) [13], and overall improved quality of life [14]. However, not all studies have yielded positive outcomes [[15], [16], [17]], possibly due to variations in patient inclusion criteria, treatment duration, and n–3 PUFA dosage. Although several traditional pairwise meta-analyses investigated the benefits of n–3 PUFA supplementation in heart failure [8,9,[18], [19], [20], [21], [22], [23]], they have not provided a comprehensive analysis of the time–dose relationship, largely due to methodologic limitations. In our previous large-scale network meta-analysis (NMA) in cardiology, we observed that the effectiveness of n–3 PUFAs may vary considerably based on dosage and treatment duration [24]. Furthermore, traditional pairwise meta-analyses fail to offer detailed insights into the comparative and superior efficacy of different n–3 PUFA supplementation regimens in patients with heart failure.

Although previous studies have examined the effectiveness of n–3 PUFAs in heart failure, their results have been inconsistent due to methodologic challenges, including differences in dosage, treatment duration, and patient characteristics. To date, no NMA has been conducted to comprehensively evaluate the effects of various n–3 PUFA supplementation protocols on heart failure outcomes. This NMA aimed to synthesize the existing evidence and provide a clearer understanding of the optimal n–3 PUFA supplementation strategy for patients with heart failure.

## Methods

This NMA followed the guidelines set forth by the PRISMA extension for NMAs [25] (Supplemental Table 1). The study was registered in PROSPERO under registration number CRD42024590476, and ethical approval was obtained from the Institutional Review Board of the Tri-Service General Hospital, National Defense Medical Center, Taipei, Taiwan (TSGHIRB No. E202416045). This study did not direct involve individual participant so that we did not have the chance to approach individual participant or explore individual participant’s information. Therefore, it would be impossible to obtain consent to publish in this study.

### Database searches and study identification

We performed comprehensive searches across multiple databases, including PubMed, Embase, ClinicalKey, Cochrane CENTRAL, ProQuest, ScienceDirect, Web of Science, and clinicaltrials.gov (Supplemental Table 2). The systematic review and NMA search commenced on 13 September, 2024. Two authors (P-TT and B-YZ) independently carried out electronic searches, screened titles and abstracts, and resolved eligibility disputes through consensus. Additionally, manual searches were performed by reviewing reference lists of relevant review articles and meta-analyses [8,9,[18], [19], [20], [21], [22], [23]]. No language restrictions were applied to the search.

### Inclusion and exclusion criteria

The NMA adhered to the PICOS model (Population, Intervention, Comparison, Outcome, and Study) with the following inclusion criteria: *1*) population: human participants diagnosed with heart failure; *2*) intervention: supplementation with n–3 PUFAs; *3*) comparison: control groups receiving either standard medication or placebo; *4*) outcome: changes in target outcomes as defined in the Outcome Definition section; and *5*) study design: RCTs.

To ensure the robustness of this NMA, only RCTs meeting the following criteria were included: *1*) RCTs involving patients with heart failure (to reduce heterogeneity, only those based on NYHA classification or equivalent criteria were included); *2*) RCTs evaluating the efficacy of n–3 PUFA supplementation; and *3*) human clinical trials. The exclusion criteria were as follows: *1*) non-RCT studies; *2*) RCTs not enrolling patients with heart failure; *3*) RCTs not comparing n–3 PUFA supplementation; *4*) RCTs not reporting the predefined outcomes; *5*) RCTs lacking specific information on the EPA/DHA ratio, as our aim was to compare the efficacy of different EPA/DHA ratios; and *6*) animal studies.

### Methodologic quality appraisal

Two independent authors evaluated risk of bias for each domain using the Cochrane Risk of Bias tool 1.0 [26], achieving an interrater reliability score of 0.85. Any disagreements were resolved by a third author.

### Outcome definition

The primary outcome in this NMA was the assessment of heart function, specifically focusing on changes in LVEF. Secondary outcomes included peak VO_2_, blood concentrations of BNP, and quality of life. The safety profile was evaluated by monitoring dropout rates (ie, participants who left the study before completion for any reason) and all-cause mortality.

### n–3 PUFA supplementation definition

The experimental groups were categorized based on the dosage and the ratio of EPA to DHA. Dosages were classified into the following groups: low (<1000 mg/d), medium (≥1000 but <2000 mg/d), high (≥2000 but < 4000 mg/d), and Extreme_high (≥4000 mg/d). Additionally, we classified the groups based on EPA/DHA ratios into pure_DHA (only DHA), DHA_dominant (EPA/DHA < 1), Equal (EPA/DHA = 1), EPA_dominant (EPA/DHA > 1), and pure_EPA (only EPA).

### Data extraction, management, and conversion

Two authors (P-TT and B-YZ) independently extracted data, including demographic details, study design, treatment characteristics, and primary and secondary outcomes from the reviewed studies. In cases where essential data were missing, the corresponding authors were contacted. Data extraction and handling followed the guidelines set forth in the Cochrane Handbook for Systematic Reviews of Interventions and relevant medical literature [27].

### Statistical analyses

Given the presence of multiple treatment arms, a random-effects model was applied to the NMA [28] using MetaInsight (version 4.0.2; Complex Reviews Support Unit, National Institute for Health Research, London, United Kingdom) within a frequentist framework. MetaInsight is a web-based platform for NMA that incorporates the netmeta package in R software, enabling frequentist statistical calculations [29].

Initially, forest plots were generated to display the SMD along with its 95% CIs for continuous outcomes (e.g., changes in LVEF, peak VO_2_, BNP concentrations, and quality of life), as well as the odds ratios (ORs) with their 95% CIs for categorical outcomes (e.g., dropout rate and all-cause mortality) [30]. Each treatment was subsequently ranked, and effect sizes from both direct and indirect comparisons were compiled in tables. To evaluate the consistency between direct and indirect treatment effect estimates, we used a node splitting technique. This method separates evidence for a specific comparison (node) into direct and indirect components and is ideal for NMA when trial-level data are available [29,31]. Statistical significance was defined as a 2-tailed *P* value of <0.05.

### Sensitivity analyses

To further evaluate the stability of our findings, we performed sensitivity analyses by grouping RCTs based on treatment duration. Specifically, we categorized treatment duration into short-term (i.e., <1 y) and long-term (i.e., 1 y or more).

### General declaration

This study complies with the principles outlined in the tenets of the Declaration of Helsinki.

## Results

### Eligibility of the studies

**FIGURE 1:**
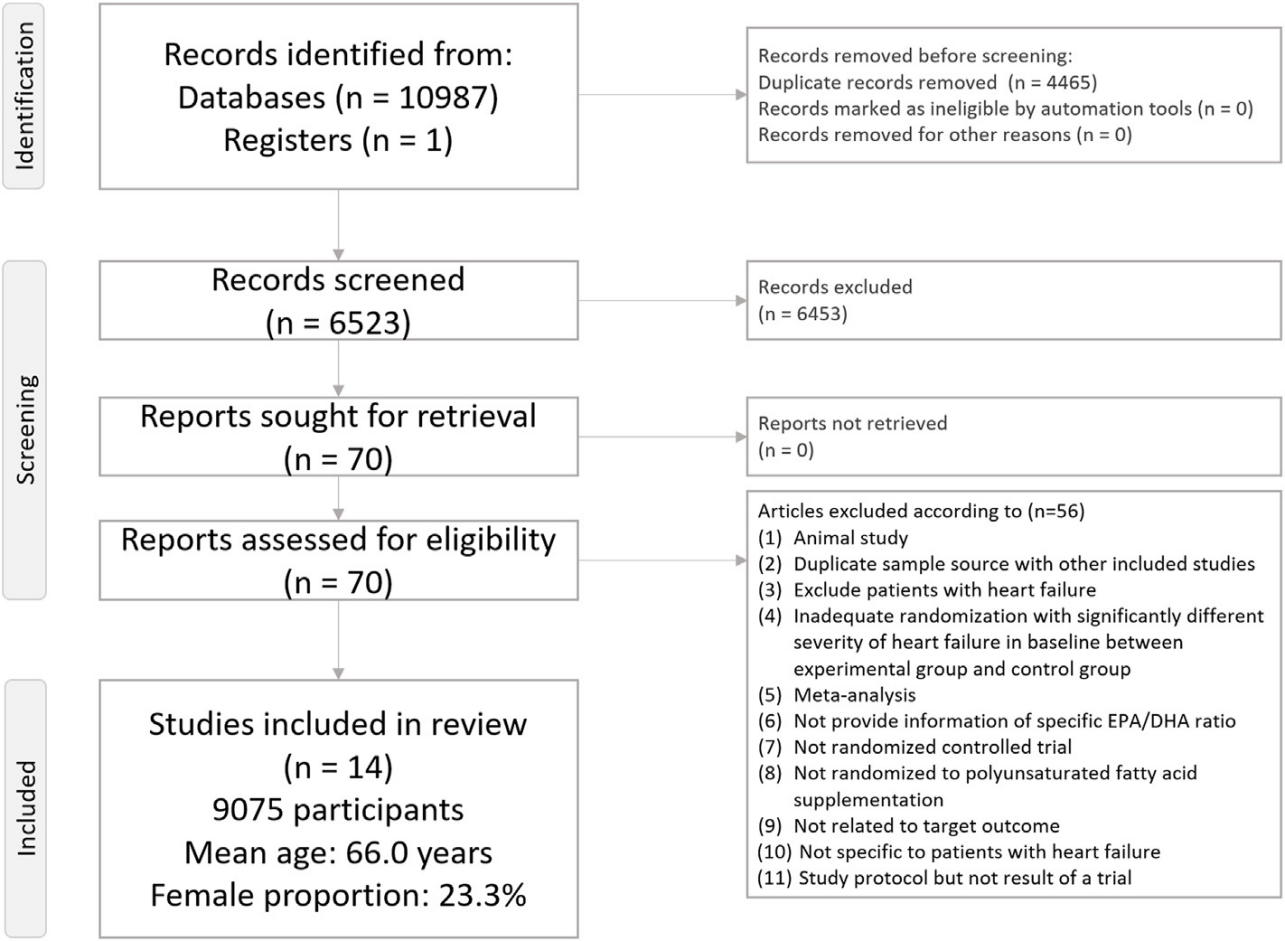
PRISMA2020 flowchart of current network meta-analysis.

Figure 1 presents the flowchart of the literature search and screening process for this NMA. After excluding 56 articles for various reasons (Supplemental Table 3) [8,9,12,15,[18], [19], [20], [21], [22], [23],[32], [33], [34], [35], [36], [37], [38], [39], [40], [41], [42], [43], [44], [45], [46], [47], [48], [49], [50], [51], [52], [53], [54], [55], [56], [57], [58], [59], [60], [61], [62], [63], [64], [65], [66], [67], [68], [69], [70], [71], [72], [73], [74], [75], [76], [77]], 14 RCTs were included. The included studies comprised 9075 participants (mean age: 66.0; range: 48.0–75.5 y; mean female proportion: 23.3%; range: 4.0%–59.3%) (Table 1) [3,10,11,13,14,[78], [79], [80], [81], [82], [83], [84], [85], [86]]. The mean study duration was 49.6 wk (range: 4 wk to ≤6 y). A total of 9 experimental arms were evaluated (1 placebo/control arm and 8 n–3 PUFA supplementation arms).

**TABLE 1:** Characteristics of the included studies.

Study name	Baseline disease	Resources	Comparison	Subjects	Mean age (y)	Female proportion (%)	Study duration	Country
Hearon et al.: exercise [79]	Patients with a history of heart failure	Prescription medicine (fish oil)	Lovaza 2000 mg once daily (EPA/DHA = 465/375) Placebo (1.6 g/d olive oil)	13 16	50.0 ± 6.0 50.0 ± 6.0	61.5 50.0	52 wk	United States
Hearon et al.: no exercise [79]	Patients with a history of heart failure	Prescription medicine (fish oil)	Lovaza 2000 mg once daily (EPA/DHA = 465/375) Placebo (1.6 g/d olive oil)	14 13	47.0 ± 9.0 49.0 ± 6.0	71.4 46.2	52 wk	United States
Selvaraj et al. (REDUCE-IT) [85]	Patients with a history of heart failure	Prescription medicine (fish oil)	Icosapent ethyl 4000 mg daily (pure EPA) Placebo	703 743	63.0	30.7	≤6 y	Multiple countries
Bonilla Palomas et al. [78]	Patients with clinical evidence of heart failure	Not specified resource	n–3 PUFA 4000 mg once daily (EPA/DHA = 460/380) Placebo (gelatin)	20 23	76.0 ± 7.7 75.0 ± 9.6	30.0 26.1	4 wk	Spain
Oikonomou et al. [83]	Patients with symptoms of heart failure	Not specified resource	n–3 PUFA 2000 mg once daily (EPA/DHA = 46%/38%) Placebo (2.0 g/d olive oil)	15 16	66.0 ± 4.0 67.0 ± 7.0	30.0 26.0	8 wk	Multiple countries
Jiang et al. (OCEAN) [80]	Patients with chronic heart failure	Fish oil supplement	n–3 PUFA 2000 mg daily (EPA/DHA = 400/200) n–3 PUFA 2000 mg daily (pure EPA) Placebo (corn oil)	36 36 36	57.7 ± 16.1 58.1 ± 10.2 57.9 ± 11.7	41.7 55.6 63.9	12 wk	United States
Wurm et al. [86]	Patients with chronic heart failure	Not specified resource	n–3 PU = 465/375)n–3 PUFA 4000 mg once daily (EPA/DHA = 465/375) Placebo (gelatin)	12 12 16	59.0 64.0 56.0	16.7 0.0 25.0	12 wk	Austria
Makarewicz-Wujec et al. [3]	Patients with a history of heart failure	Fish oil supplement	n–3 PUFA 2000 mg daily (EPA/DHA = 255/179) Placebo (2.0 g/d corn oil)	15 15	67.5 ± 0.7 62.1 ± 1.4	13.3 13.3	12 wk	Poland
Wu et al. [14]	Patients with a history of heart failure	Fish oil supplement	n–3 PUFA 6500 mg daily (EPA/DHA = 3285/3285) Placebo (safflower oil)	14 12	59.0 ± 3.0 56.0 ± 2.0	14.3 25.0	12 wk	United States
Moertl et al. [81]	Patients with chronic heart failure and reduced left ventricular ejection fraction	Prescription medicine (fish oil)	Omacor 1000 mg once daily (EPA/DHA = 465/375)Omacor 4000 mg once daily (EPA/DHA = 465/375) Placebo (gelatin)	14 13 16	58.6 ± 7.0 61.9 ± 9.6 55.1 ± 12.7	14.3 0.0 25.0	12 wk	Austria
Nodari et al. [13]	Patients with chronic heart failure	Prescription medicine (fish oil)	Omacor 2000 mg daily (EPA/DHA = 0.9/1.5) Placebo (1.0 g/d olive oil)	67 66	61.0 ± 11.0 64.0 ± 9.0	4.5 15.2	52 wk	United States
Marchioli et al. (GISSI-HF, part of R1) [10]	Patients with clinical evidence of heart failure	Fish oil supplement	n–3 PUFA 1000 mg once daily (EPA/DHA = 1.2:1) Placebo	3494 3481	67.0 ± 11.0 67.0 ± 11.0	22.2 21.2	52 wk	Italy
Nodari et al. [82]	Patients with heart failure	Not specified resource	n–3 PUFA 1000 mg daily (EPA/DHA = 0.9/1.5) Placebo (1.0 g/d olive oil)	22 22	61.1 ± 11.2 64.8 ± 9.5	4.5 13.6	24 wk	United States
Zhao et al. [11]	Patients with symptoms of heart failure	Not specified resource	n–3 PUFA 2000 mg once daily (EPA/DHA = 180/120) Placebo	38 37	74.0 ± 6.0 71.0 ± 10.0	29.0 24.0	12 wk	China
Radaelli et al. [84]	Patients with chronic heart failure	Fish oil supplement	n–3 PUFA 2000 mg daily (EPA/DHA = 0.9/1.5) Placebo	15 10	59.4 ± 2.5 60.1 ± 2.7	NA	16 wk	Italy

Abbreviations: NA: not available.

Among the included studies, there were no any RCTs using n–3 PUFAs with resources of dietary, algae, or krill. Most of them used n–3 PUFAs of fish oil extracts or prescription medicine (Table 1). All n–3 PUFAs were daily token in oral form. None, 4, 7, and 5 of them prescribed low dosage (<1000 mg/d), medium dosage (≥1000 but <2000 mg/d) [10,81,82,86], high dosage (≥2000 but <4000 mg/d) [3,11,13,79,80,83,84], and extreme high dosage (≥4000 mg/d) [14,78,81,85,86] of n–3 PUFA supplement, respectively. All included participants had a history of heart failure.

### Primary outcome: changes of LVEF

**FIGURE 2:**
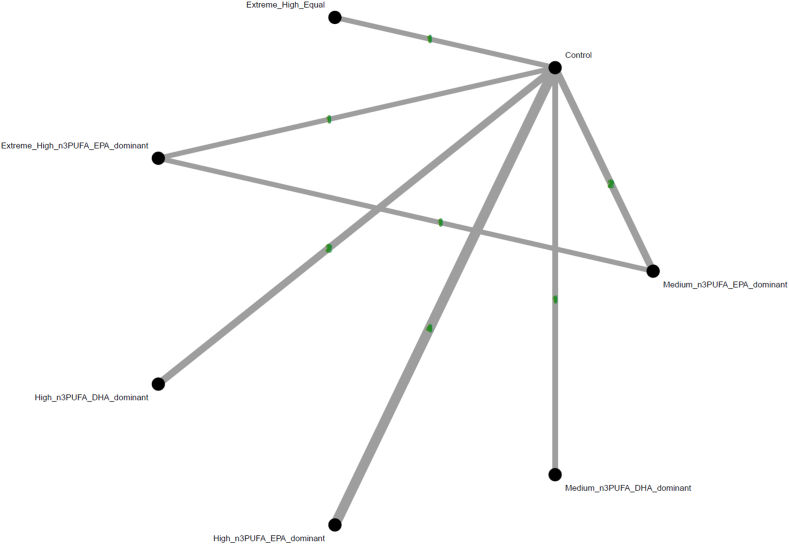
Network structure of the primary outcome: changes of left ventricular ejection fraction (LVEF). The overall structure of the network meta-analysis. The lines between nodes represent direct comparisons from various trials, with the numbers over the lines indicating the number of trials providing these comparisons for each specific treatment. The thickness of the lines corresponds to the number of trials linked to the network. Extreme_High_Equal, extreme high dosage (≥4000 mg/d) n–3PUFA treatment with composition of EPA/DHA ratio equal to 1; Extreme_High_n3PUFA_EPA_dominant, extreme high dosage (≥4000 mg/d) n–3PUFA treatment with composition of EPA predominant; Extreme_High_pure_EPA, extreme high dosage (≥4000 mg/d) n–3PUFA treatment with composition of pure EPA; High_n3PUFA_DHA_dominant, high dosage (≥2000 mg/d but <4000 mg/d) n–3PUFA treatment with composition of DHA predominant; High_n3PUFA_EPA_dominant, high dosage (≥2000 mg/d but <4000 mg/d) n–3PUFA treatment with composition of EPA predominant; High_pure_EPA, high dosage (≥2000 mg/d but <4000 mg/d) n–3PUFA treatment with composition of pure EPA; Medium_n3PUFA_DHA_dominant, medium dosage (≥1000 mg/d but <2000 mg/d) n–3PUFA treatment with composition of DHA predominant; Medium_n3PUFA_EPA_dominant, medium dosage (≥1000 mg/d but <2000 mg/d) n–3PUFA treatment with composition of EPA predominant; NMA, network meta-analysis; OR, odds ratio.

**FIGURE 3:**
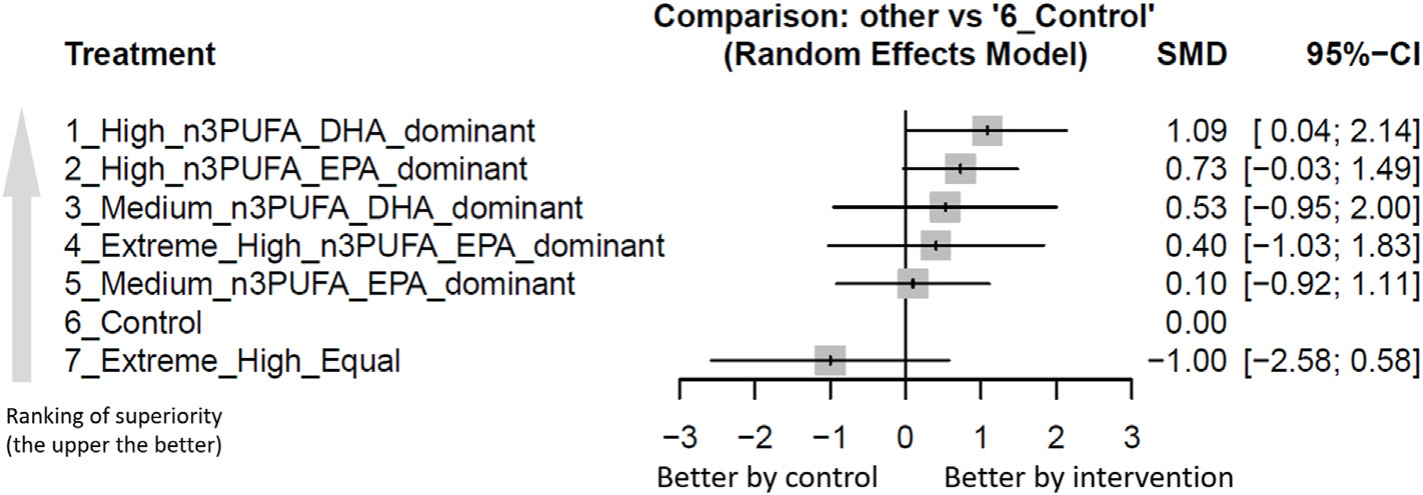
Forest plot of primary outcome: changes of left ventricular ejection fraction (LVEF). When the effect size (expressed as SMDs) was >0, the specified treatment was associated with greater improvement in LVEF in patients with heart failure than in patients receiving controls. Further, we marked the ranking of superiority of each experimental arm. Extreme_High_Equal, extreme high dosage (≥4000 mg/d) n–3PUFA treatment with composition of EPA/DHA ratio equal to 1; Extreme_High_n3PUFA_EPA_dominant, extreme high dosage (≥4000 mg/d) n–3PUFA treatment with composition of EPA predominant; Extreme_High_pure_EPA, extreme high dosage (≥4000 mg/d) n–3PUFA treatment with composition of pure EPA; High_n3PUFA_DHA_dominant, high dosage (≥2000 mg/d but <4000 mg/d) n–3PUFA treatment with composition of DHA predominant; High_n3PUFA_EPA_dominant, high dosage (≥2000 mg/d but <4000 mg/d) n–3PUFA treatment with composition of EPA predominant; High_pure_EPA, high dosage (≥2000 mg/d but <4000 mg/d) n–3PUFA treatment with composition of pure EPA; Medium_n3PUFA_DHA_dominant, medium dosage (≥1000 mg/d but <2000 mg/d) n–3PUFA treatment with composition of DHA predominant; Medium_n3PUFA_EPA_dominant, medium dosage (≥1000 mg/d but <2000 mg/d) n–3PUFA treatment with composition of EPA predominant; NMA, network meta-analysis; OR, odds ratio.

Only the high dosage of n–3 PUFAs with DHA predominant (SMD: 1.09; 95% CI: 0.04, 2.14) was associated with a significantly more improvement in LVEF than the control group did. Among these interventions, high dosage of n–3 PUFAs with DHA predominant ranked the best, whereas high dosage of n–3 PUFAs with EPA predominant (SMD: 0.73; 95% CI: –0.03, 1.49; in comparison with controls) the second (FIGURE 2, FIGURE 3, and Table 2).

**TABLE 2:** League table of the primary outcome: changes of left ventricular ejection fraction.

High_n3PUFA_DHA_dominant					1.09 (0.04, 2.14)^1^	
0.36 (−0.94, 1.65)	High_n3PUFA_EPA_dominant				0.73 (−0.03, 1.49)	
0.56 (−1.25, 2.37)	0.20 (−1.46, 1.86)	Medium_n3PUFA_DHA_dominant			0.53 (−0.95, 2.00)	
0.68 (−1.09, 2.46)	0.32 (−1.29, 1.94)	0.12 (−1.93, 2.18)	Extreme_High_n3PUFA_EPA_dominant	0.28 (−1.26, 1.83)	0.43 (−1.11, 1.96)	
0.99 (−0.47, 2.45)	0.63 (−0.63, 1.90)	0.43 (−1.36, 2.22)	0.31 (−1.13, 1.74)	Medium_n3PUFA_EPA_dominant	0.10 (−0.92, 1.11)	
1.09 (0.04, 2.14)^1^	0.73 (−0.03, 1.49)	0.53 (−0.95, 2.00)	0.40 (−1.03, 1.83)	0.10 (−0.92, 1.11)	Control	1.00 (−0.58, 2.58)
2.09 (0.19, 3.98)^1^	1.73 (−0.02, 3.48)	1.53 (−0.64, 3.69)	1.40 (−0.72, 3.53)	1.10 (−0.78, 2.97)	1.00 (−0.58, 2.58)	Extreme_High_Equal

Data present as SMD (95% CIs). Pairwise (upper-right portion) and network (lower-left portion) meta-analysis results are presented as estimate effect sizes for the outcome of changes of LVEF in patients with heart failure. Interventions are reported in order of mean ranking of beneficial effect on improvement of LVEF, and outcomes are expressed as SMD (95%CIs). For the pairwise meta-analyses, SMD of >0 indicate that the treatment specified in the row got more beneficial effect than that specified in the column. For the NMA, SMD of >0 indicate that the treatment specified in the column got more beneficial effect than that specified in the row.

Abbreviations: Extreme_High_Equal, extreme high dosage (≥4000 mg/d) n–3PUFA treatment with composition of EPA/DHA ratio equal to 1; Extreme_High_n3PUFA_EPA_dominant, extreme high dosage (≥4000 mg/d) n–3PUFA treatment with composition of EPA predominant; Extreme_High_pure_EPA, extreme high dosage (≥4000 mg/d) n–3PUFA treatment with composition of pure EPA; High_n3PUFA_DHA_dominant, high dosage (≥2000 mg/d but <4000 mg/d) n–3PUFA treatment with composition of DHA predominant; High_n3PUFA_EPA_dominant, high dosage (≥2000 mg/d but <4000 mg/d) n–3PUFA treatment with composition of EPA predominant; High_pure_EPA, high dosage (≥2000 mg/d but <4000 mg/d) n–3PUFA treatment with composition of pure EPA; Medium_n3PUFA_DHA_dominant, medium dosage (≥1000 mg/d but <2000 mg/d) n–3PUFA treatment with composition of DHA predominant; Medium_n3PUFA_EPA_dominant, medium dosage (≥1000 mg/d but <2000 mg/d) n–3PUFA treatment with composition of EPA predominant; NMA, network meta-analysis; OR, odds ratio.

1 Statistical significance.

#### Sensitivity analysis of primary outcome by subgroup analysis of short-term treatment compared with long-term treatment

The results of subgroup of short-term treatment (i.e., <1 y) revealed that none of the investigated n–3 PUFA supplement exerted significantly different efficacy in the changes of LVEF in patients with heart failure from those in controls (Supplemental Figures 1A and 2A, and Supplemental Table 4A).

On the contrary, the results of subgroup of long-term treatment (i.e., ≥1 y) revealed that both high dosage of n–3 PUFAs with DHA predominant (SMD: 2.12; 95% CI: 1.70, 2.55) and high dosage of n–3 PUFAs with EPA predominant (SMD: 0.67; 95% CI: 0.13, 1.21) were associated with significantly more improvement in LVEF than that in the control. Among these interventions, high dosage of n–3 PUFAs with DHA predominant ranked the best, whereas high dosage of n–3 PUFAs with EPA predominant the second (Supplemental Figures 1B and 2B, and Supplemental Table 4B).

### Secondary outcome: changes of peak VO_2_

The medium dosage of n–3 PUFAs with DHA predominant (SMD: 1.10; 95% CI: 0.47, 1.74) extreme high dosage of n–3 PUFAs with EPA predominant (SMD: 0.84; 95% CI: 0.09, 1.59), and high dosage of n–3 PUFAs with DHA predominant (SMD: 0.78; 95% CI: 0.42, 1.13) were associated with significantly more improvement in peak VO_2_ than that in the control group did. Among these interventions, medium dosage of n–3 PUFAs with DHA predominant ranked the best, followed by extreme high dosage of n–3 PUFAs with EPA predominant and high dosage of n–3 PUFAs with DHA predominant (Supplemental Figures 1C and 2C, and Supplemental Table 4C).

### Secondary outcome: changes of blood BNP concentrations

None of the n–3 PUFA supplementation arms showed significant changes in blood BNP concentrations compared with the control group (Supplemental Figures 1D and 2D, and Supplemental Table 4D).

### Secondary outcome: changes of quality of life

In this part of analysis, there were 2 RCTs providing information on the changes of the quality of life. Among them, Wu et al. [14] used Kansas City Cardiomyopathy Questionnaire overall score, and Jiang et al. [80] applied General Health Questionnaire. All of them evaluated quality of life from an overall view but not from specific aspects in specific field. No significant differences were observed in quality of life outcomes between the n–3 PUFA supplementation groups and the control group (Supplemental Figures 1E and 2E, and Supplemental Table 4E).

### Safety profile: dropout rate

None of the n–3 PUFA supplementation arms were associated with significantly different dropout rates compared with the control group (Supplemental Figures 1F and 2F, and Supplemental Table 4F).

### Safety profile: all-cause mortality

No significant differences were found in all-cause mortality between the n–3 PUFA supplementation groups and the control group (Supplemental Figures 1G and 2G, and Supplemental Table 4G).

### Risk of bias and inconsistency

We identified that 87.8% (86/98 items), 12.2% (12/98 items), and 0.0% (0/98 items) of the included studies had low, unclear, and high risks of bias, respectively (Supplemental Figure 3A,B). The inconsistency test, which assesses the assumption of consistency, revealed no significant inconsistencies in this NMA (Supplemental Table 5).

## Discussion

To our knowledge, this NMA was the first to comprehensively evaluate the comparative efficacy of different dosages and treatment durations of n–3 PUFA supplementation in patients with heart failure. The most significant finding of this study is the time-dependent and dosage-dependent benefits of n–3 PUFA supplementation on heart failure outcomes, particularly improvements in LVEF and peak VO_2_. Notably, in RCTs with long-term treatments (i.e., ≥1 y), both high dosage n–3 PUFAs with either EPA or DHA predominance led to significantly greater improvements in LVEF than the control treatment. Additionally, none of the treatment groups showed significant differences in dropout rates or all-cause mortality when compared with controls.

Previous traditional pairwise meta-analyses attempted to explore the effects of n–3 PUFA supplementation on heart failure outcomes [8,9,[18], [19], [20], [21], [22], [23]]. However, these studies did not provide a comprehensive analysis of the time–dose relationship, likely due to methodologic limitations. As highlighted in our previous large-scale NMAs and related studies [24,[87], [88], [89], [90], [91], [92]], the beneficial effects of n–3 PUFA supplementation exhibit clear time and dose dependency, particularly with higher doses and longer treatment durations across various diseases.

n–3 PUFA supplementation has demonstrated cardioprotective mechanisms in heart failure, including anti-inflammatory properties, improved endothelial function, and regulation of autonomic nervous system activity [93]. Additionally, animal studies have shown that n–3 PUFAs promote both diastolic and systolic function recovery and reduce adverse ventricular remodeling [94,95]. This NMA further suggests that the positive effects of n–3 PUFAs on heart function and ventricular remodeling only occur with higher doses (≥2000 mg/d) and longer durations (≥1 y). Repairing adverse ventricular remodeling during heart failure is a key factor in reducing mortality, sudden cardiac death, and the incidence of heart failure [8]. This repair process is gradual and cannot be achieved through short-term treatments, underscoring the importance of long-term treatment in clinical trials focused on heart failure outcomes.

One potential explanation for the beneficial effects of n–3 PUFAs on heart function and ventricular remodeling lies in their anti-inflammatory, antioxidant, and membrane-stabilizing properties [8,9]. As previous studies have noted, the induction of specific cytokines, such as IL-6 and TNF-α, plays a detrimental role in the development of adverse ventricular remodeling and subsequent heart failure [96], contributing to conditions such as cardiac hypertrophy and impaired myocardial contractility. The relationship between ventricular dysfunction and oxidative stress is primarily driven by several mechanisms [9] as follows: *1*) oxidation of thiol groups in ryanodine receptor Ca^2+^ channels, leading to abnormal channel opening [97]; *2*) mitochondrial damage, resulting in cardiac cell apoptosis and ventricular decompensation [98]; and *3*) abnormal cardiac remodeling, fibroblast proliferation, and matrix remodeling [9,99]. Some of these negative pathways can be mitigated or reversed through antioxidant treatment [100]. Further, they might be distinctly mitigated by various DHA/EPA ratio [46]. Several studies have reported that n–3 PUFA supplementation reduces inflammatory markers in patients with heart failure [65,81], including TNF-α, IL–1, and IL–6 [101]. Consequently, by reducing oxidative stress, heart function can be improved, and heart failure symptoms alleviated [9,81]. Finally, reports had proven a dose-dependent benefit on the amelioration of ventricular remodeling and myocardial fibrosis by various dosage of n–3 PUFA supplement [93].

### Strengths and limitations

Our NMA has several strengths. First, it provides comprehensive comparative evidence regarding the time-dependent and dosage-dependent efficacy of n–3 PUFA supplementation in patients with heart failure, which traditional pairwise meta-analyses could not achieve. Second, to enhance the reliability of our findings, only RCTs were included, excluding non-RCTs and case–control studies. Third, we minimized heterogeneity by focusing on RCTs that specifically enrolled patients with a definitive diagnosis of heart failure.

However, this NMA also has some limitations. First, some analyses may have been underpowered due to variability in treatment characteristics, such as study duration. To address this, we conducted subgroup analyses based on treatment duration, distinguishing between short-term (<1 y) and long-term (≥1 y) treatments. Among the included RCTs, only 1 RCT provided result of efficacy of n–3 PUFA supplementation >1 y—that is, ≤6 y in the RCT by Selvaraj et al. [85]—which reported insignificant benefit on all-cause mortality by n–3 PUFAs. Therefore, the efficacy of n–3 PUFA supplementation beyond 1 y remains unclear. Moreover, the heterogeneity among the recruited participants in the included RCTs, such as acute compared with chronic heart failure, wide range of ages, gender distribution, and single compared with multiple centers, might have potential bias in this study. Second, our strict inclusion criteria excluded non-RCTs, which may have limited the number of studies included in this NMA. Further, the restrictive exclusion criteria regarding the necessary information of EPA/DHA ratio would limit the number of studies included in this study. For example, the report by Kojuri et al. [54] was excluded due to lack of information regarding EPA/DHA ratio, in which report the authors noticed high dosage n–3 PUFAs (2000 mg/d) could significantly reduce BNP concentration and improve outcomes of 6-min walking test.

### Conclusions

Long-term, high dosage n–3 PUFA supplementation significantly enhances heart failure outcomes, particularly in improving LVEF and peak VO_2_, without raising dropout rates or all-cause mortality. These findings advocate for the inclusion of specific n–3 PUFAs regimens in the management of heart failure. Further well-designed RCTs with extended treatment durations (ie, beyond 1 y) are recommended to validate these results.

## Author contributions

The authors’ responsibilities were as follows – P-TT, B-YZ, C-WH: contributed equally as first authors and took the whole responsibility of literature search, data extraction, data analysis, and manuscript writing; C-SL, BS, Y-WC, T-YC, W-TL, J-JC: contributed to study design, concept formation, and manuscript revision; P-TT, Y-LS, K-PS: who contributed equally as corresponding authors, took the whole responsibility of collection of information from the other authors, final content of manuscript major revision, and manuscript submission; and all authors: have read and approved the manuscript.

## Funding

The authors reported no funding received for this study.

## Data availability

The data of this study would be available upon reasonable request to the corresponding authors.

## Conflict of interest

BS is supported by the NIHR Brendon Stubbs is part funded by the NIHR Biomedical Research Centre at South London and Maudsley NHS Foundation Trust. BS is also supported by the Maudsley Charity, King’s College London. All other authors report no conflicts of interest.
