# Supplementary material for: The Optimal Dosage and Duration of ω-3 PUFA Supplementation in Heart Failure Management: Evidence from a Network Meta-Analysis
Source: Adv Nutr. 2025 Jan 11;16(2):100366. doi: 10.1016/j.advnut.2025.100366 (PMC11836506; doi:10.1016/j.advnut.2025.100366)
Supplement: multimedia component 1 [file mmc1.docx]

**List of content in supplement materials**

**The Optimal Dosage and Duration of Omega-3 Polyunsaturated Fatty Acid Supplementation in Heart Failure Management:**

**Evidence from a Network Meta-Analysis**

Ping-Tao Tseng, et al.

| Supplemental Figure 1 | (A) network structure of NMA of subgroup of changes of LVEF in short-term treatment duration |
| --- | --- |
|  | (B) network structure of NMA of subgroup of changes of LVEF in long-term treatment duration |
|  | (C) network structure of NMA of changes of peak VO_2_ |
|  | (D) network structure of NMA of changes of blood BNP levels |
|  | (E) network structure of NMA of changes of quality of life |
|  | (F) network structure of NMA of drop-out rate |
|  | (G) network structure of NMA of all-cause mortality |
| Supplemental Figure 2 | (A) forest plot of NMA of subgroup of changes of LVEF in short-term treatment duration |
|  | (B) forest plot of NMA of subgroup of changes of LVEF in long-term treatment duration |
|  | (C) forest plot of NMA of changes of peak VO_2_ |
|  | (D) forest plot of NMA of changes of blood BNP levels |
|  | (E) forest plot of NMA of changes of quality of life |
|  | (F) forest plot of NMA of drop-out rate |
|  | (G) forest plot of NMA of all-cause mortality |
| Supplemental Figure 3 | (A) overview of risk of bias |
|  | (B) detailed risk of bias in each study |
| Supplemental Table 1 | PRISMA 2020 checklist of the current network meta-analysis |
| Supplemental Table 2 | Keyword used in each database and search result |
| Supplemental Table 3 | Excluded studies and reason |
| Supplemental Table 4 | (A): League table of the subgroup of changes of LVEF in short-term treatment duration |
|  | (B): League table of the subgroup of changes of LVEF in long-term treatment duration |
|  | (C): League table of the secondary outcome: changes of changes of peak VO_2_ |
|  | (D): League table of the secondary outcome: changes of blood BNP levels |
|  | (E): League table of the secondary outcome: changes of quality of life |
|  | (F): League table of the safety profile: drop-out rate |
|  | (G): League table of the safety profile: all-cause mortality |
| Supplemental Table 5 | inconsistency within the network meta-analysis of primary outcome: changes of left ventricular ejection fraction |

**Supplemental Figure 1A network structure of NMA of subgroup of changes of LVEF in short-term treatment duration**

**
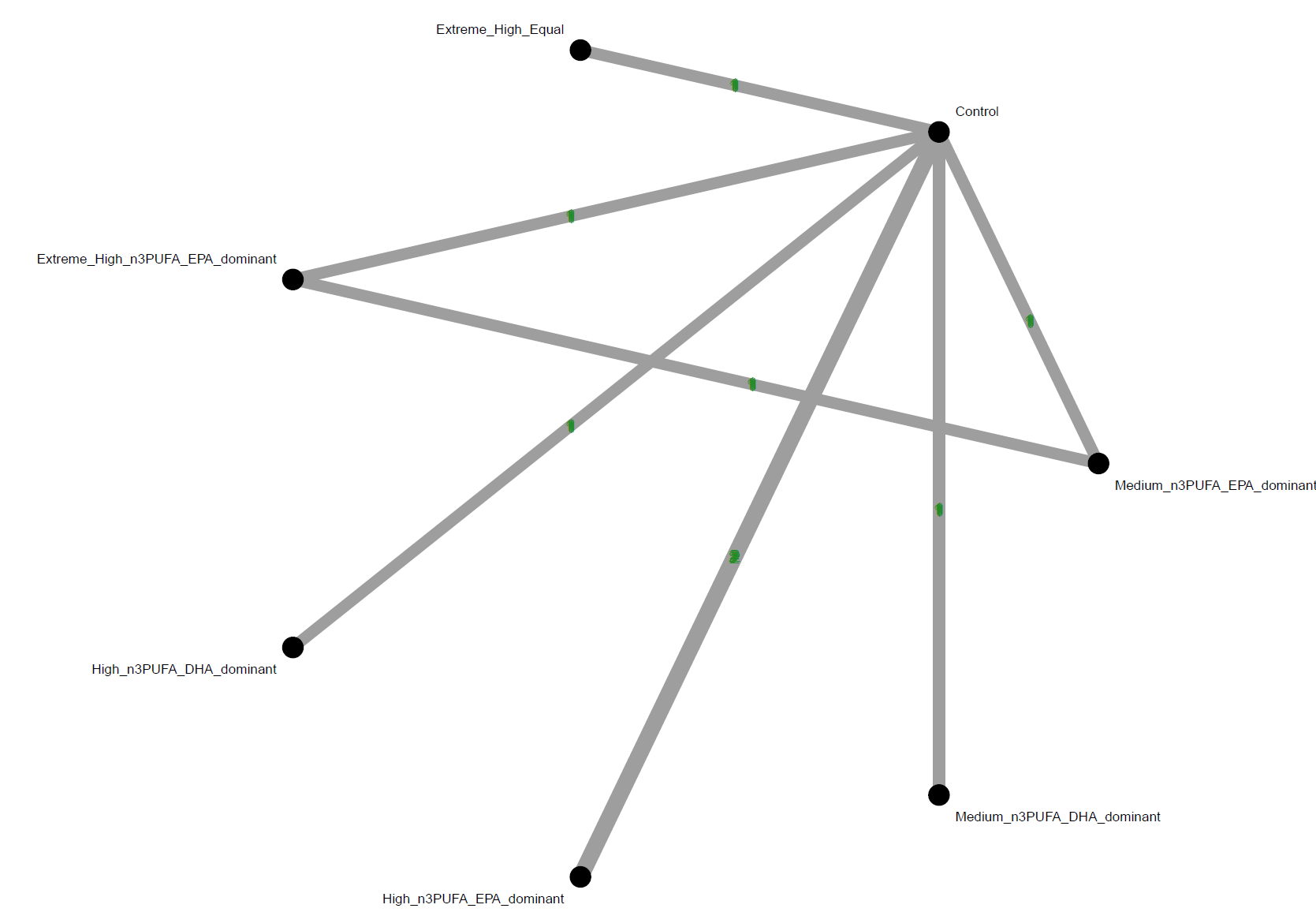
**

**Supplemental Figure 1B network structure of NMA of subgroup of changes of LVEF in long-term treatment duration**

**
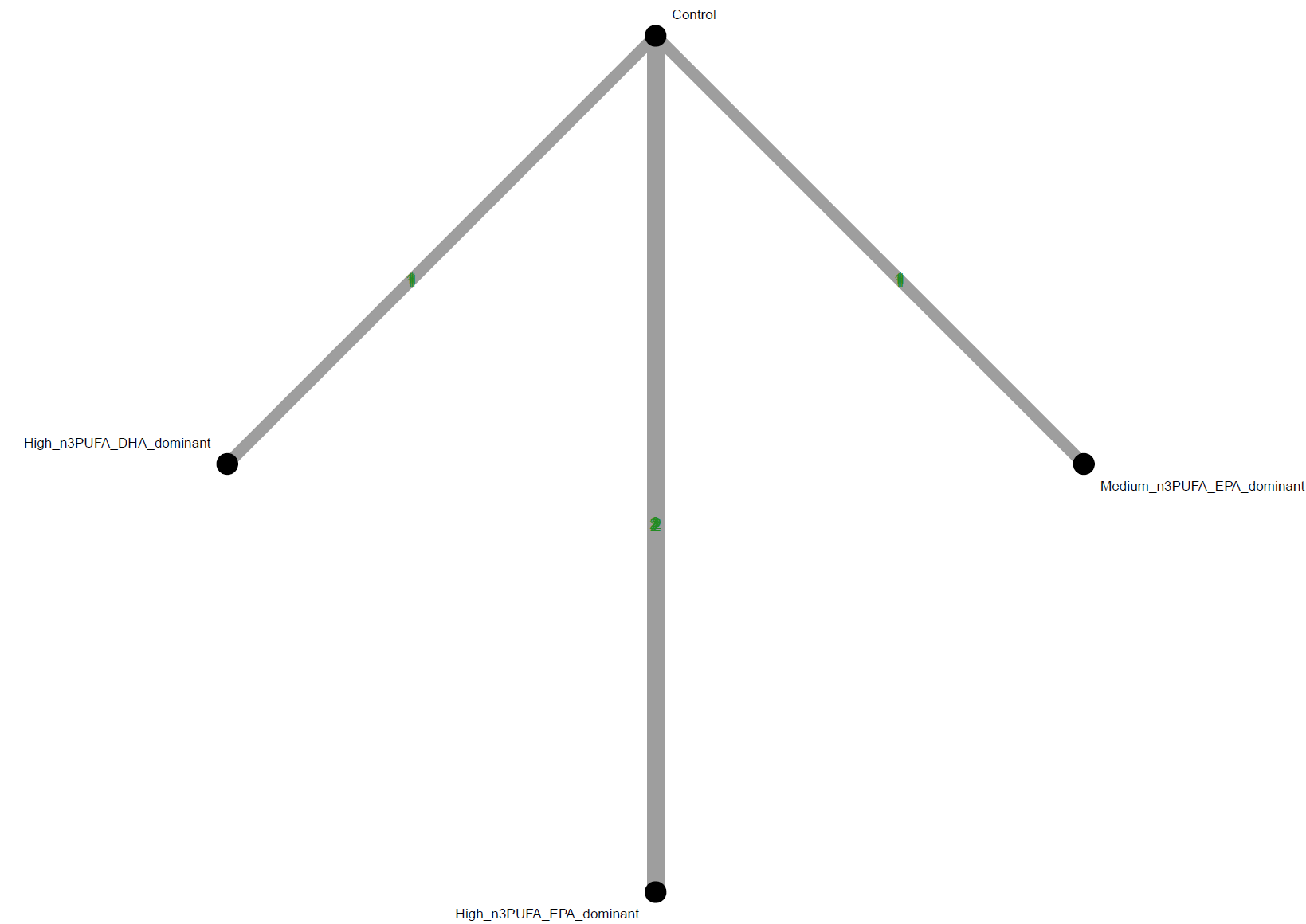
**

**Supplemental Figure 1C network structure of NMA of changes of peak VO_2_**

**
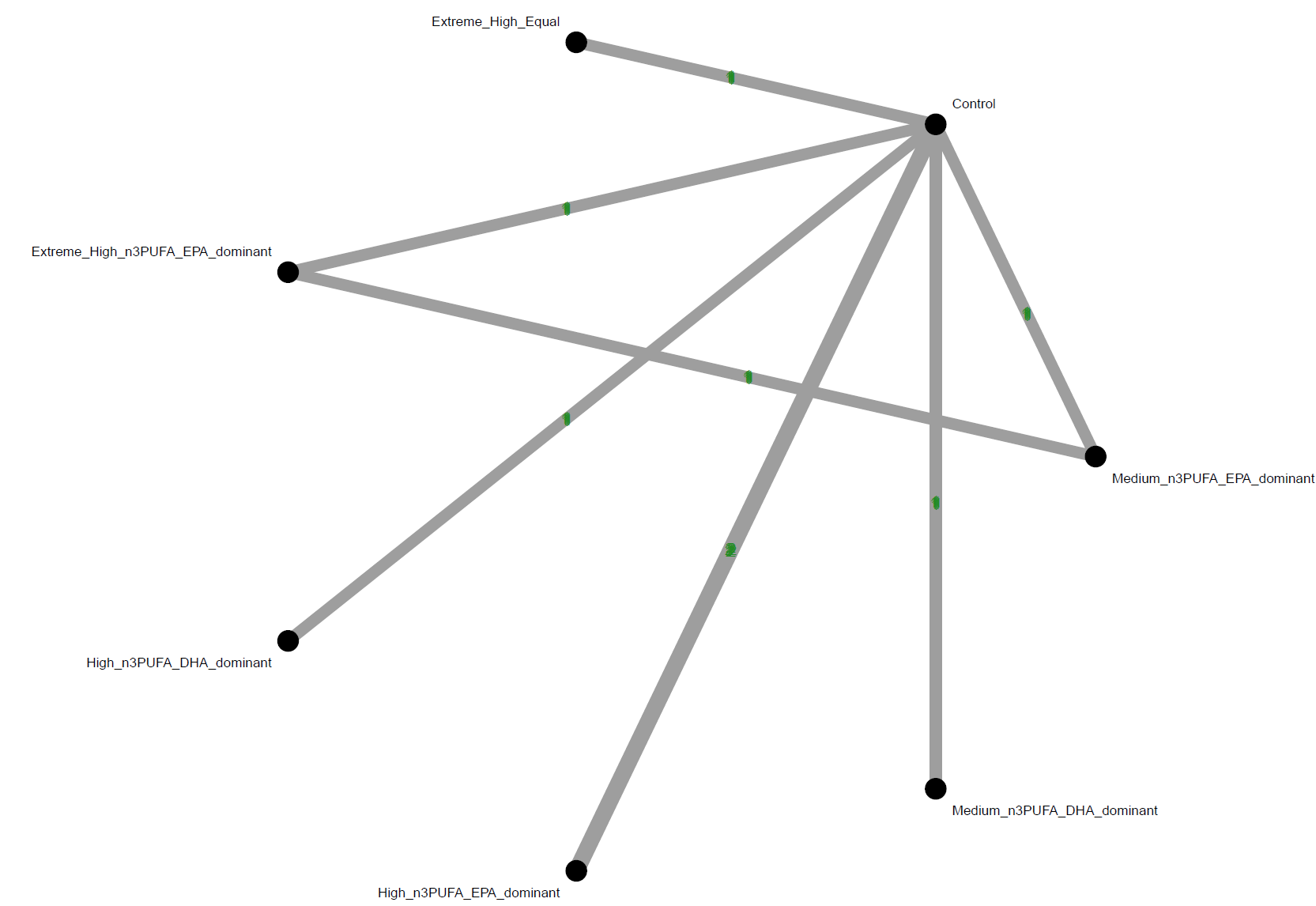
**

**Supplemental Figure 1D network structure of NMA of changes of blood BNP levels**

**
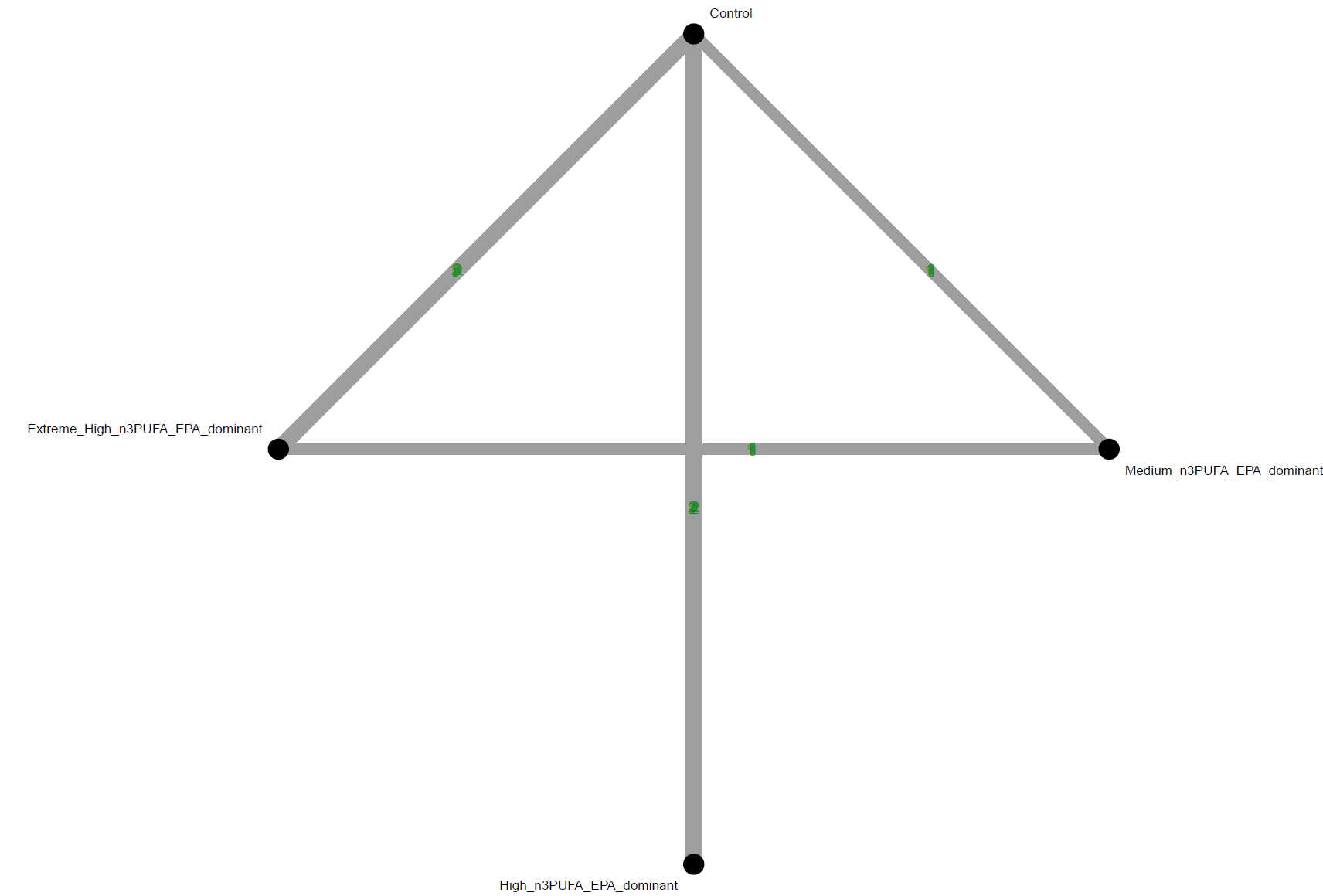
**

**Supplemental Figure 1E network structure of NMA of changes of quality of life**

**
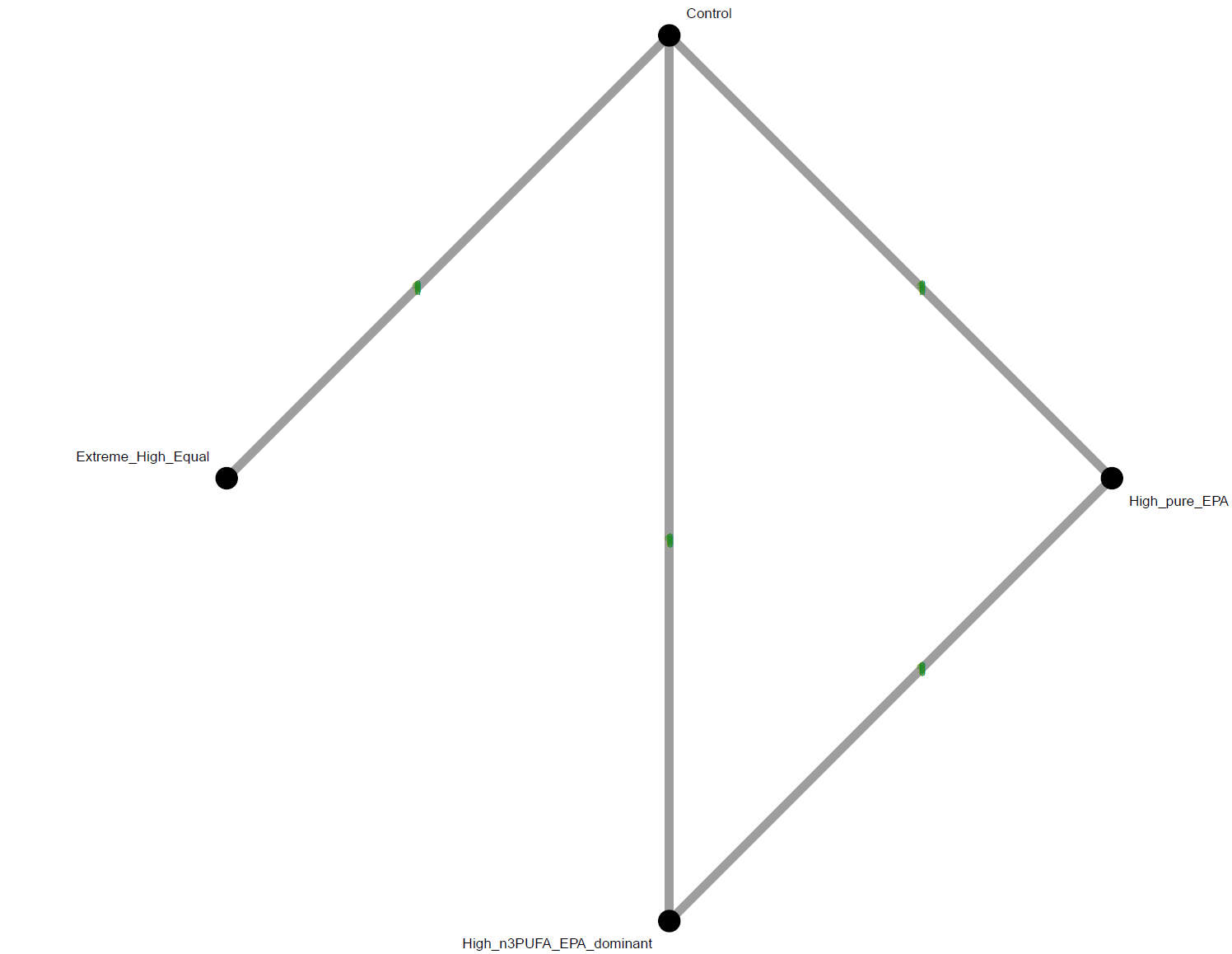
**

**Supplemental Figure 1F network structure of NMA of drop-out rate**

**
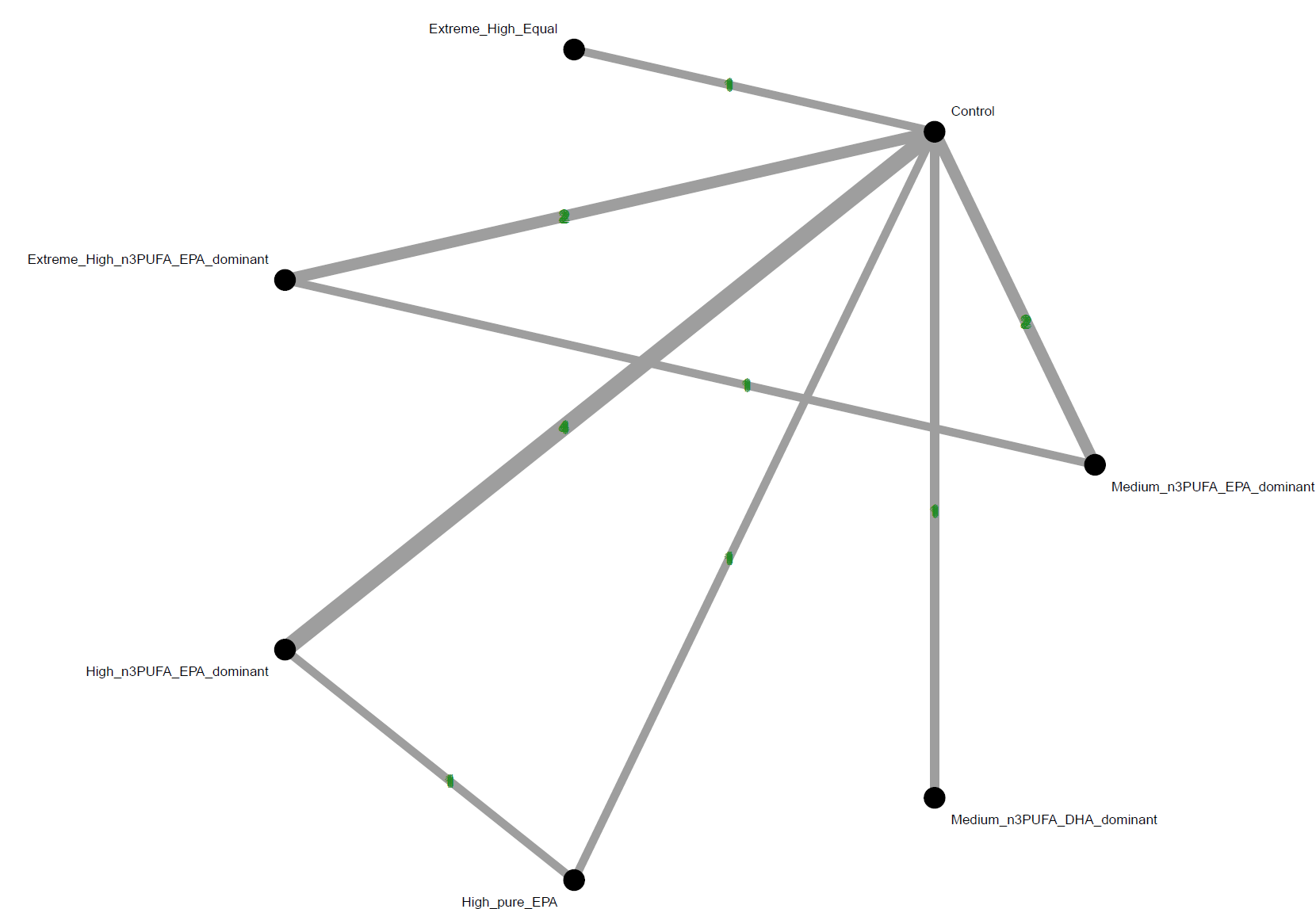
**

**Supplemental Figure 1G network structure of NMA of all-cause mortality**

**
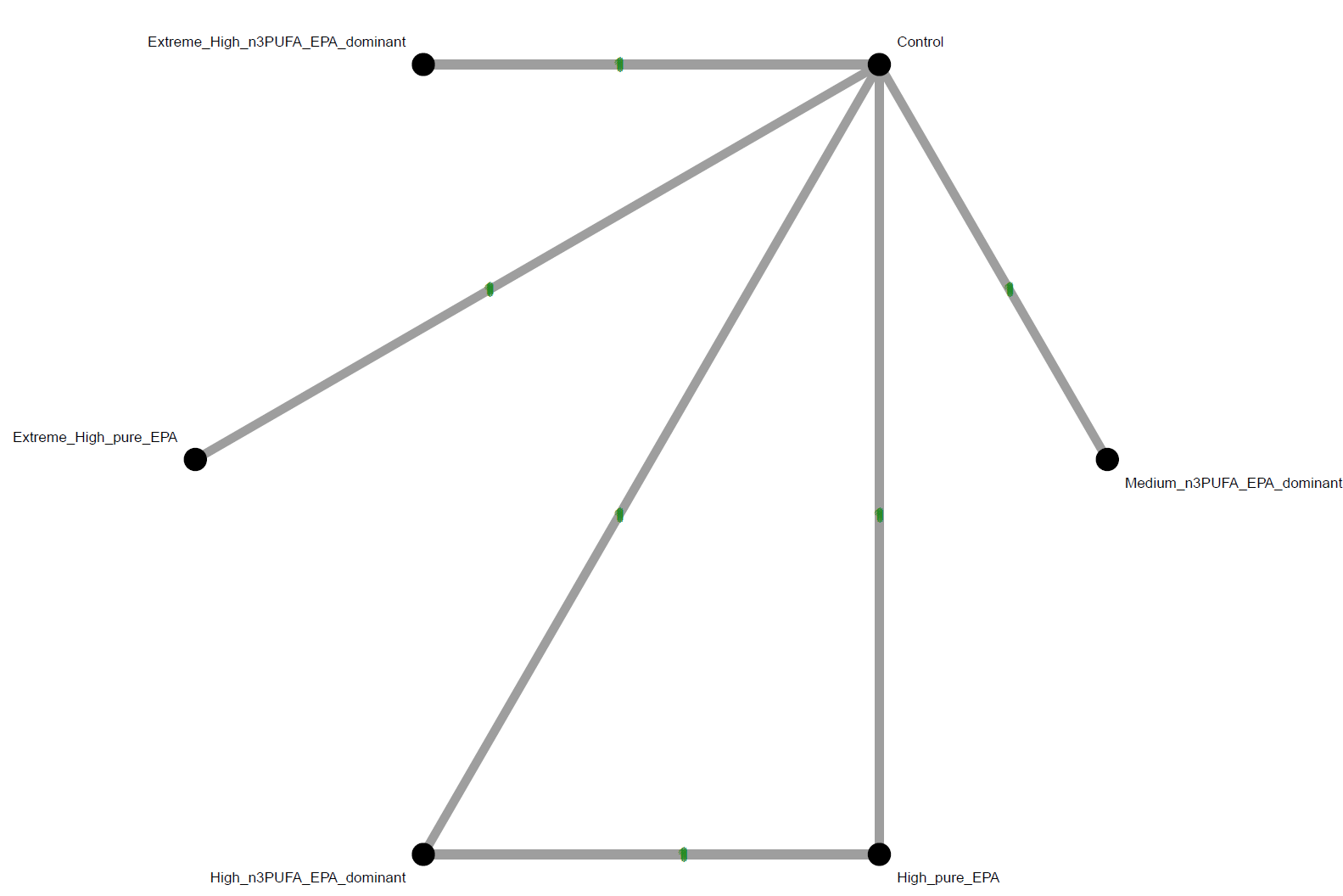
**

**Figure legend of Supplemental Figure 1A-1G**

The overall structure of the network meta-analysis. The lines between nodes represent direct comparisons from various trials, with the numbers over the lines indicating the number of trials providing these comparisons for each specific treatment. The thickness of the lines corresponds to the number of trials linked to the network.

*Abbreviation for Supplemental Figure 1A-1G: 95%CIs: 95% confidence intervals; BNP: brain natriuretic peptide; DHA: docosahexaenoic acid; EPA: eicosapentaenoic acid; Extreme_High_Equal: extreme high dosage (at least 4000 mg/day) n3PUFA treatment with composition of EPA/DHA ratio equal to 1; Extreme_High_n3PUFA_EPA_dominant: extreme high dosage (at least 4000 mg/day) n3PUFA treatment with composition of EPA predominant; Extreme_High_pure_EPA: extreme high dosage (at least 4000 mg/day) n3PUFA treatment with composition of pure EPA; High_n3PUFA_DHA_dominant: high dosage (at least 2000 mg/day but less than 4000 mg/day) n3PUFA treatment with composition of DHA predominant; High_n3PUFA_EPA_dominant: high dosage (at least 2000 mg/day but less than 4000 mg/day) n3PUFA treatment with composition of EPA predominant; High_pure_EPA: high dosage (at least 2000 mg/day but less than 4000 mg/day) n3PUFA treatment with composition of pure EPA; LVEF: left ventricular ejection fraction; Medium_n3PUFA_DHA_dominant: medium dosage (at least 1000 mg/day but less than 2000 mg/day) n3PUFA treatment with composition of DHA predominant; Medium_n3PUFA_EPA_dominant: medium dosage (at least 1000 mg/day but less than 2000 mg/day) n3PUFA treatment with composition of EPA predominant; n3PUFA: omega-3 polyunsaturated fatty acid; NMA: network meta-analysis; OR: odds ratio; peak VO2: peak oxygen consumption; RCT: randomized controlled trial; SMD: standardized mean difference*

**Supplemental Figure 2A forest plot of NMA of subgroup of changes of LVEF in short-term treatment duration**

**
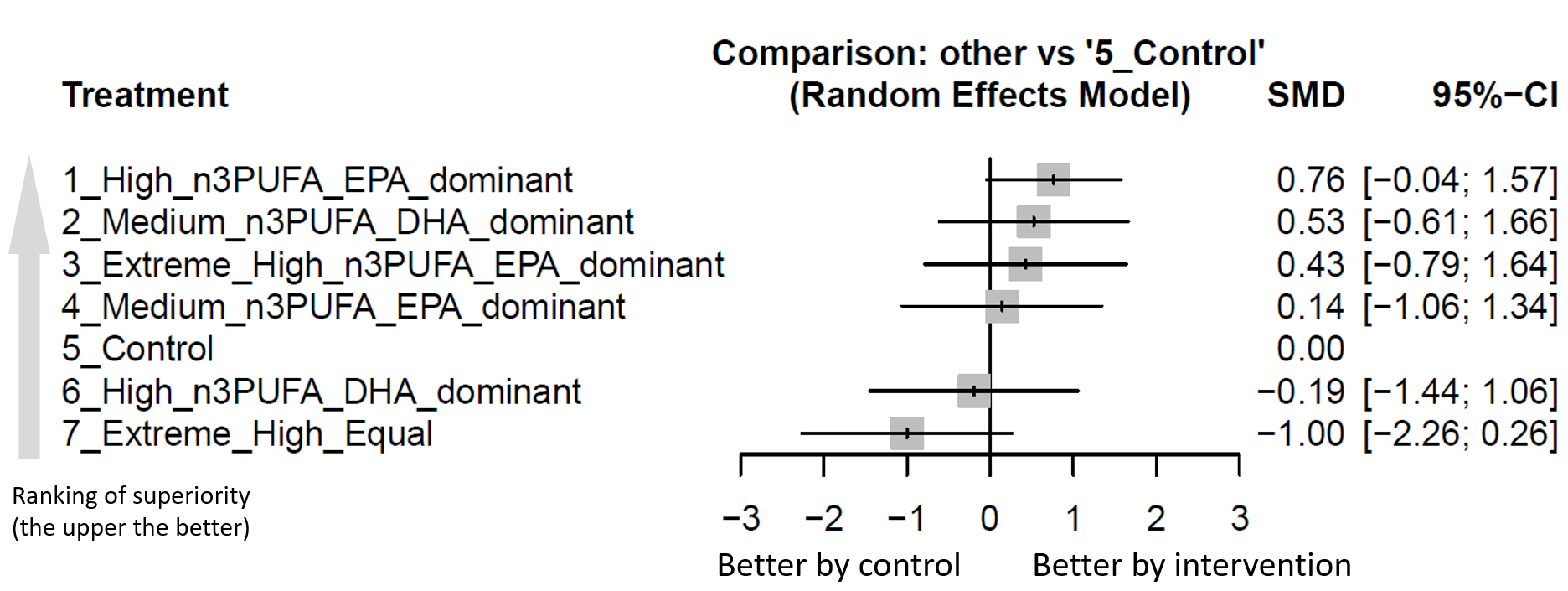
**

**Supplemental Figure 2B forest plot of NMA of subgroup of changes of LVEF in long-term treatment duration**

**
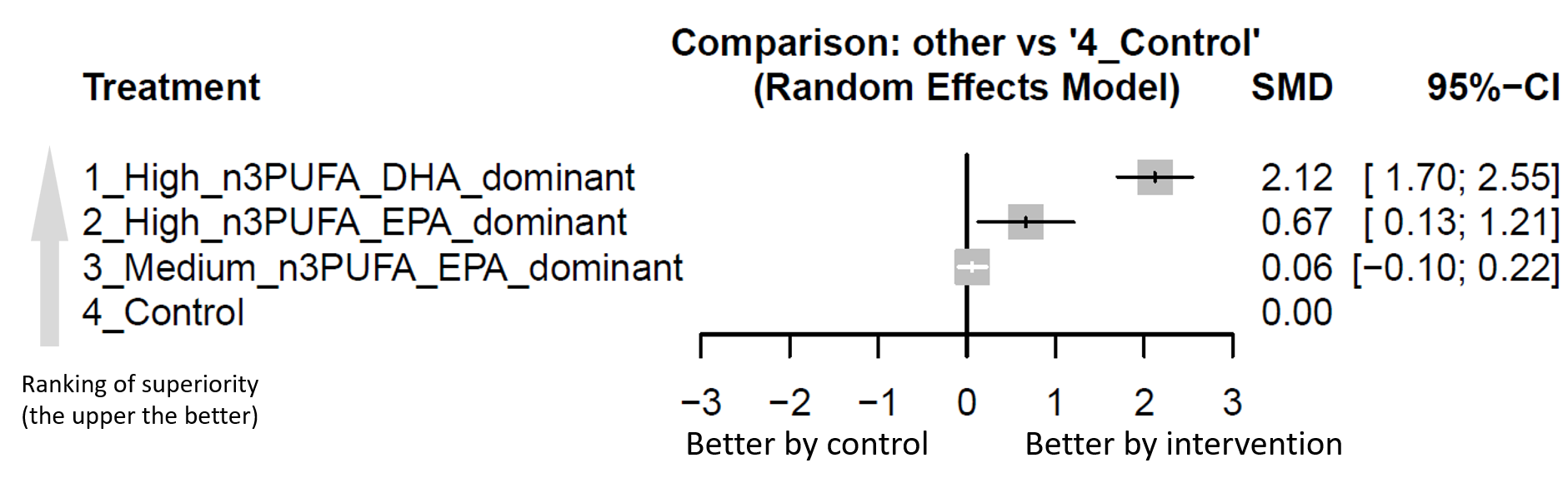
**

**Supplemental Figure 2C forest plot of NMA of changes of peak VO_2_**

**
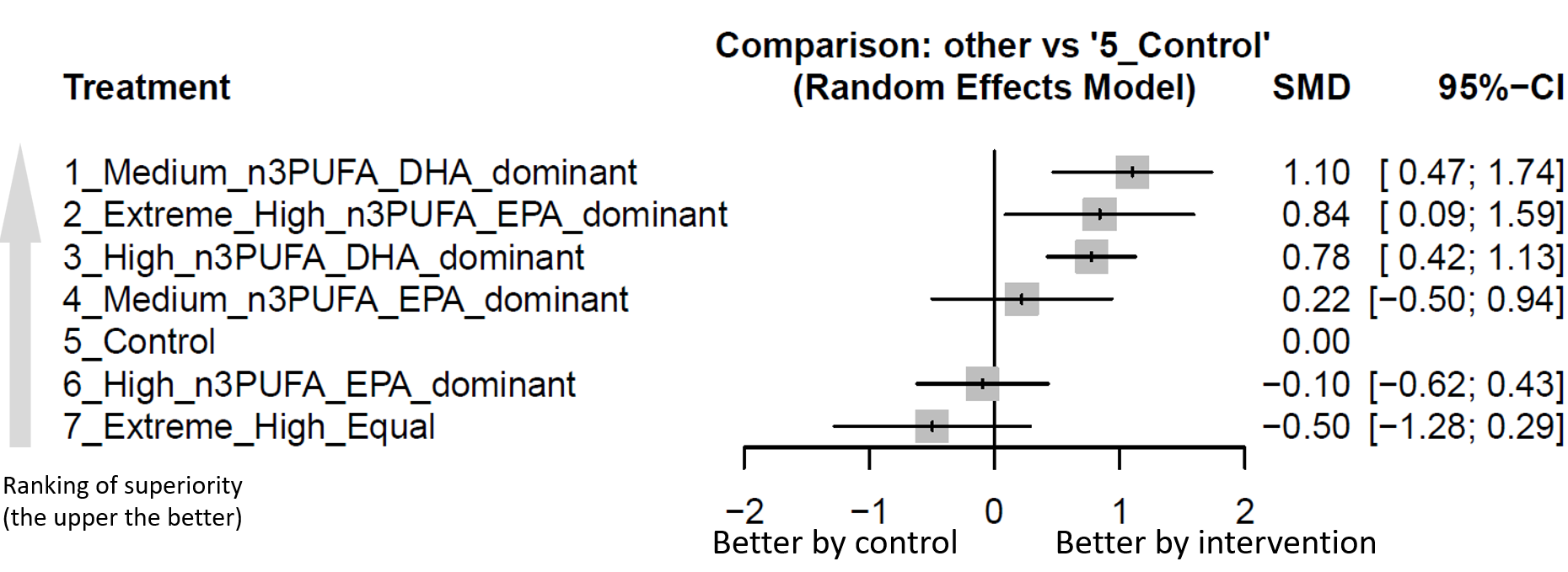
**

**Supplemental Figure 2D forest plot of NMA of changes of blood BNP levels**

**
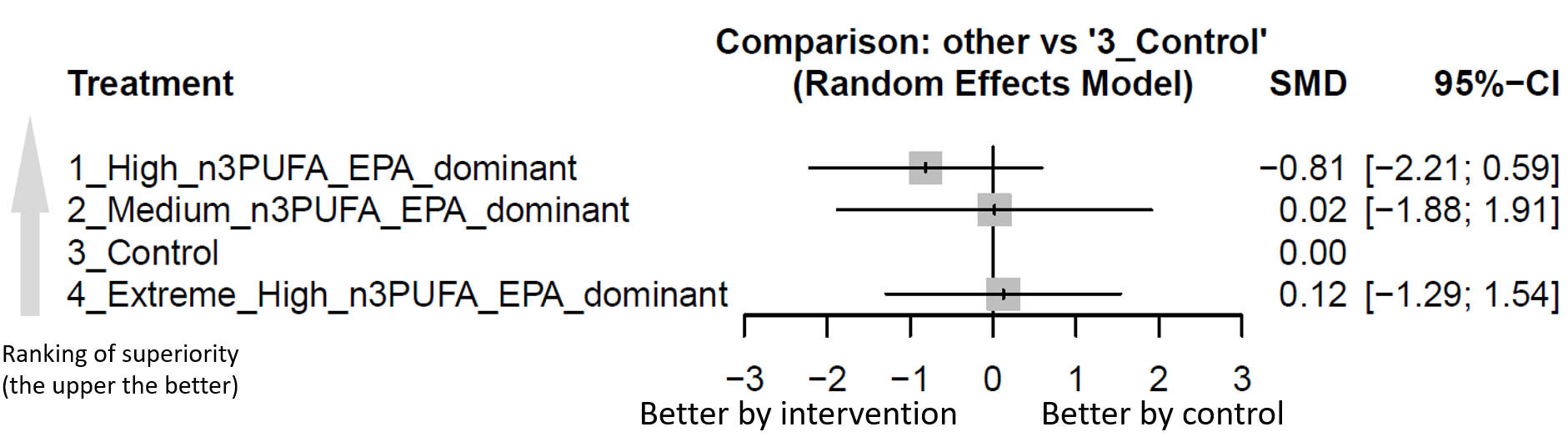
**

**Supplemental Figure 2E forest plot of NMA of changes of quality of life**

**
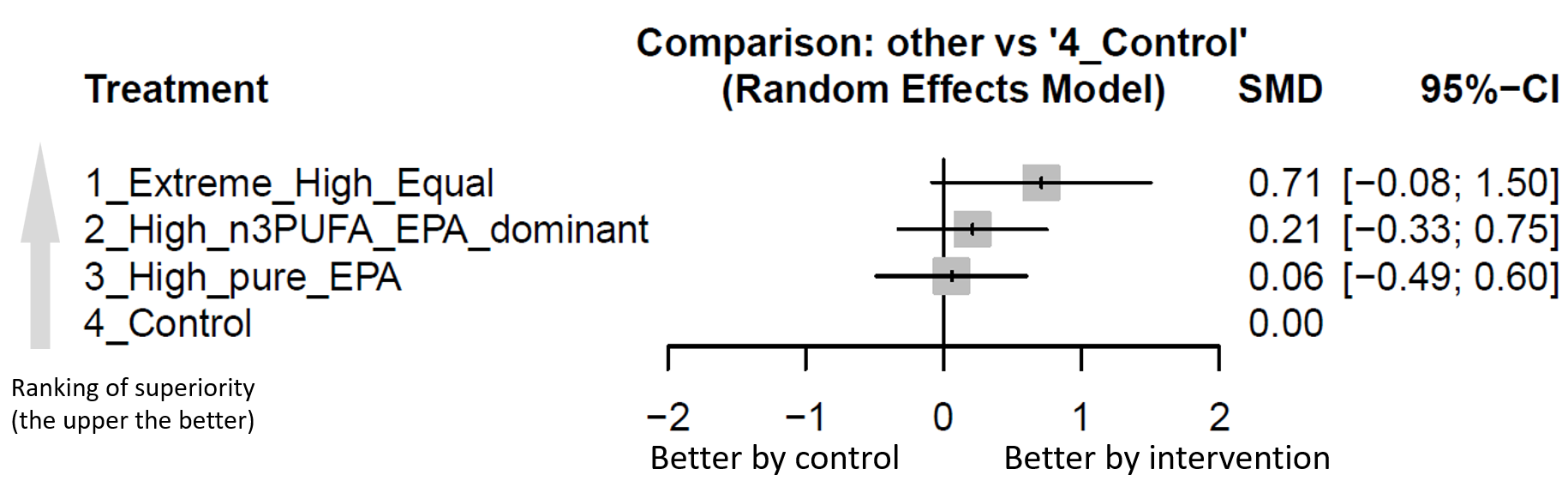
**

**Supplemental Figure 2F forest plot of NMA of drop-out rate**

**
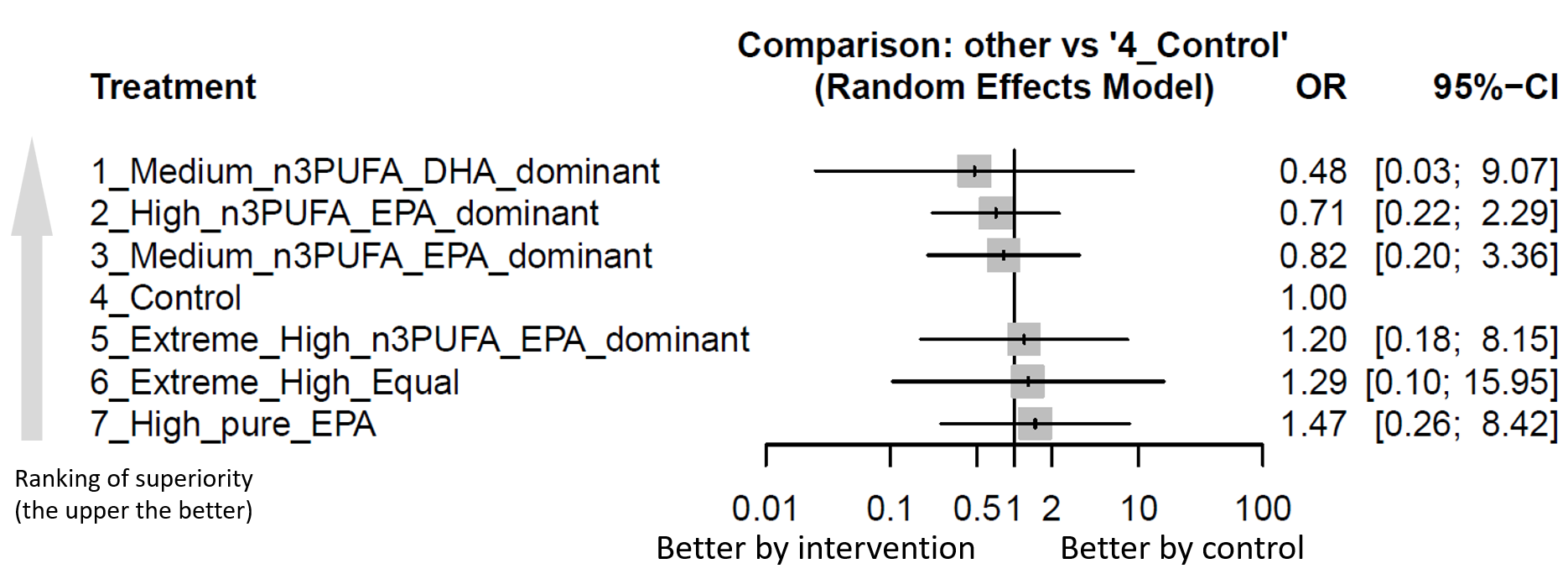
**

**Supplemental Figure 2G forest plot of NMA of all-cause mortality**

**
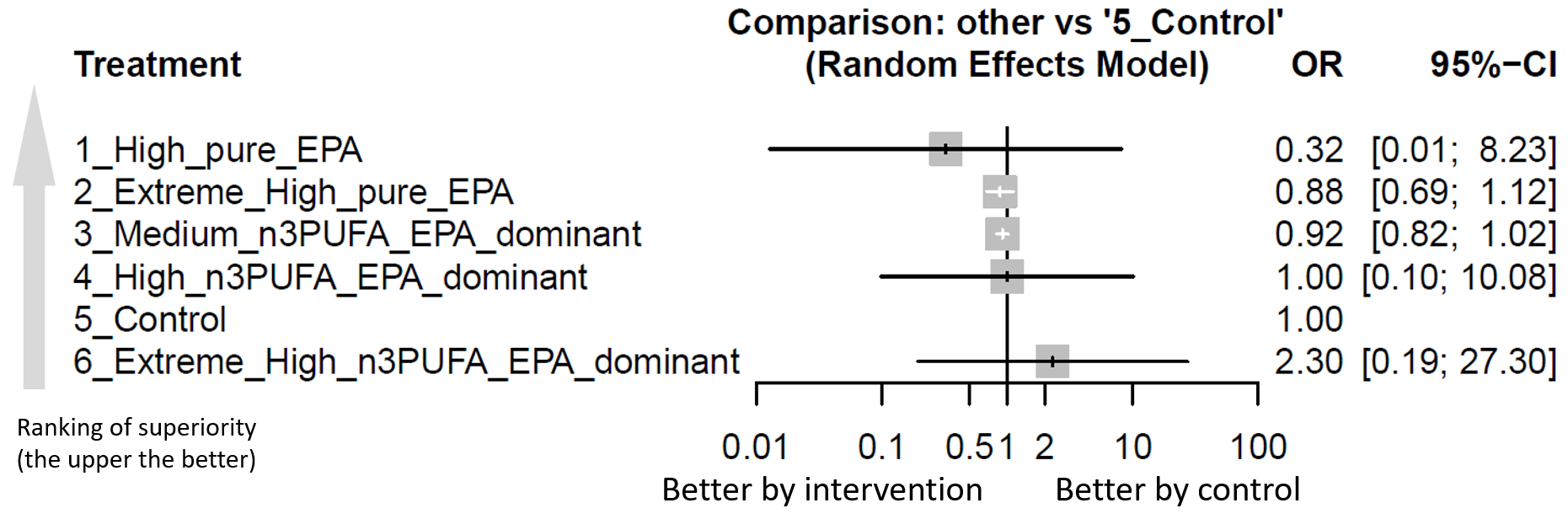
**

Abbreviation for Supplemental Figure 2A-2G: *95%CIs: 95% confidence intervals; BNP: brain natriuretic peptide; DHA: docosahexaenoic acid; EPA: eicosapentaenoic acid; Extreme_High_Equal: extreme high dosage (at least 4000 mg/day) n3PUFA treatment with composition of EPA/DHA ratio equal to 1; Extreme_High_n3PUFA_EPA_dominant: extreme high dosage (at least 4000 mg/day) n3PUFA treatment with composition of EPA predominant; Extreme_High_pure_EPA: extreme high dosage (at least 4000 mg/day) n3PUFA treatment with composition of pure EPA; High_n3PUFA_DHA_dominant: high dosage (at least 2000 mg/day but less than 4000 mg/day) n3PUFA treatment with composition of DHA predominant; High_n3PUFA_EPA_dominant: high dosage (at least 2000 mg/day but less than 4000 mg/day) n3PUFA treatment with composition of EPA predominant; High_pure_EPA: high dosage (at least 2000 mg/day but less than 4000 mg/day) n3PUFA treatment with composition of pure EPA; LVEF: left ventricular ejection fraction; Medium_n3PUFA_DHA_dominant: medium dosage (at least 1000 mg/day but less than 2000 mg/day) n3PUFA treatment with composition of DHA predominant; Medium_n3PUFA_EPA_dominant: medium dosage (at least 1000 mg/day but less than 2000 mg/day) n3PUFA treatment with composition of EPA predominant; n3PUFA: omega-3 polyunsaturated fatty acid; NMA: network meta-analysis; OR: odds ratio; peak VO2: peak oxygen consumption; RCT: randomized controlled trial; SMD: standardized mean difference*

**Supplemental Figure 3A overview of risk of bias**

**
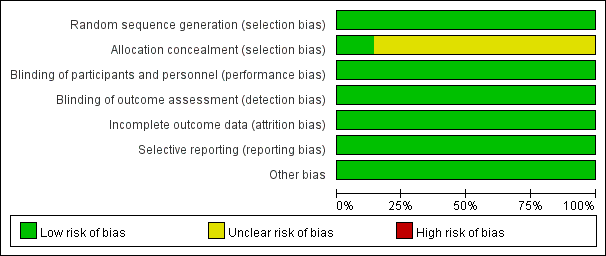
**

**Supplemental Figure 3B detailed risk of bias in each study**

**
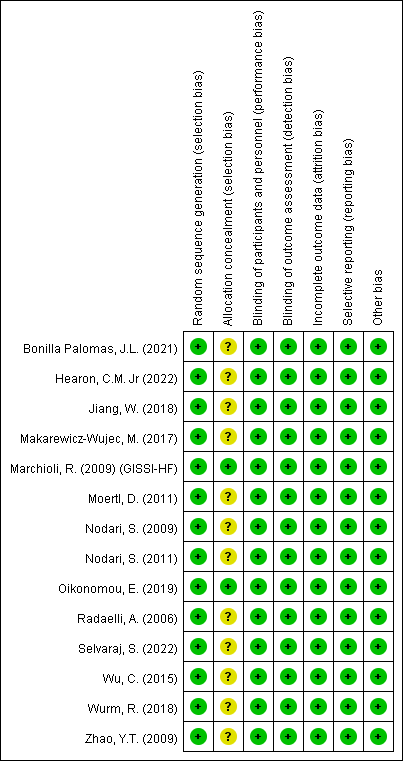
**

**Supplemental Table 1:** PRISMA 2020 checklist of the current network meta-analysis

| **Section and Topic** | **Item #** | **Checklist item** | **Page where item is reported** |
| --- | --- | --- | --- |
| **TITLE** | | |  |
| Title | 1 | Identify the report as a systematic review. | 1 |
| **ABSTRACT** | | |  |
| Abstract | 2 | See the PRISMA 2020 for Abstracts checklist. | 6 |
| **INTRODUCTION** | | |  |
| Rationale | 3 | Describe the rationale for the review in the context of existing knowledge. | 8-9 |
| Objectives | 4 | Provide an explicit statement of the objective(s) or question(s) the review addresses. | 8-9 |
| **METHODS** | | |  |
| Eligibility criteria | 5 | Specify the inclusion and exclusion criteria for the review and how studies were grouped for the syntheses. | 10-11 |
| Information sources | 6 | Specify all databases, registers, websites, organisations, reference lists and other sources searched or consulted to identify studies. Specify the date when each source was last searched or consulted. | 10-11 |
| Search strategy | 7 | Present the full search strategies for all databases, registers and websites, including any filters and limits used. | 10-11 |
| Selection process | 8 | Specify the methods used to decide whether a study met the inclusion criteria of the review, including how many reviewers screened each record and each report retrieved, whether they worked independently, and if applicable, details of automation tools used in the process. | 10-11 |
| Data collection process | 9 | Specify the methods used to collect data from reports, including how many reviewers collected data from each report, whether they worked independently, any processes for obtaining or confirming data from study investigators, and if applicable, details of automation tools used in the process. | 10-11 |
| Data items | 10a | List and define all outcomes for which data were sought. Specify whether all results that were compatible with each outcome domain in each study were sought (e.g. for all measures, time points, analyses), and if not, the methods used to decide which results to collect. | 11-12 |
|  | 10b | List and define all other variables for which data were sought (e.g. participant and intervention characteristics, funding sources). Describe any assumptions made about any missing or unclear information. | 11-12 |
| Study risk of bias assessment | 11 | Specify the methods used to assess risk of bias in the included studies, including details of the tool(s) used, how many reviewers assessed each study and whether they worked independently, and if applicable, details of automation tools used in the process. | 11-12 |
| Effect measures | 12 | Specify for each outcome the effect measure(s) (e.g. risk ratio, mean difference) used in the synthesis or presentation of results. | 11-12 |
| Synthesis methods | 13a | Describe the processes used to decide which studies were eligible for each synthesis (e.g. tabulating the study intervention characteristics and comparing against the planned groups for each synthesis (item #5)). | 11-12 |
|  | 13b | Describe any methods required to prepare the data for presentation or synthesis, such as handling of missing summary statistics, or data conversions. | 12-13 |
|  | 13c | Describe any methods used to tabulate or visually display results of individual studies and syntheses. | 12-13 |
|  | 13d | Describe any methods used to synthesize results and provide a rationale for the choice(s). If meta-analysis was performed, describe the model(s), method(s) to identify the presence and extent of statistical heterogeneity, and software package(s) used. | 12-13 |
|  | 13e | Describe any methods used to explore possible causes of heterogeneity among study results (e.g. subgroup analysis, meta-regression). | 12-13 |
|  | 13f | Describe any sensitivity analyses conducted to assess robustness of the synthesized results. | 12-13 |
| Reporting bias assessment | 14 | Describe any methods used to assess risk of bias due to missing results in a synthesis (arising from reporting biases). | 12-13 |
| Certainty assessment | 15 | Describe any methods used to assess certainty (or confidence) in the body of evidence for an outcome. | 12-13 |
| **RESULTS** | | |  |
| Study selection | 16a | Describe the results of the search and selection process, from the number of records identified in the search to the number of studies included in the review, ideally using a flow diagram. | 14-15, Fig 1, eTab 2 |
|  | 16b | Cite studies that might appear to meet the inclusion criteria, but which were excluded, and explain why they were excluded. | 14-15, eTab 3 |
| Study characteristics | 17 | Cite each included study and present its characteristics. | 14-15, Tab 1 |
| Risk of bias in studies | 18 | Present assessments of risk of bias for each included study. | 14-15, eFig 3 |
| Results of individual studies | 19 | For all outcomes, present, for each study: (a) summary statistics for each group (where appropriate) and (b) an effect estimate and its precision (e.g. confidence/credible interval), ideally using structured tables or plots. | 14-15, Tab 1 |
| Results of syntheses | 20a | For each synthesis, briefly summarise the characteristics and risk of bias among contributing studies. | 15-16, Fig 2 |
|  | 20b | Present results of all statistical syntheses conducted. If meta-analysis was done, present for each the summary estimate and its precision (e.g. confidence/credible interval) and measures of statistical heterogeneity. If comparing groups, describe the direction of the effect. | 15-16, Fig 3 |
|  | 20c | Present results of all investigations of possible causes of heterogeneity among study results. | 15-16, eTab 6 |
|  | 20d | Present results of all sensitivity analyses conducted to assess the robustness of the synthesized results. | 15-16 |
| Reporting biases | 21 | Present assessments of risk of bias due to missing results (arising from reporting biases) for each synthesis assessed. | 15-16, eFig 3 |
| Certainty of evidence | 22 | Present assessments of certainty (or confidence) in the body of evidence for each outcome assessed. | 15-16 |
| **DISCUSSION** | | |  |
| Discussion | 23a | Provide a general interpretation of the results in the context of other evidence. | 17-18 |
|  | 23b | Discuss any limitations of the evidence included in the review. | 18-19 |
|  | 23c | Discuss any limitations of the review processes used. | 18-19 |
|  | 23d | Discuss implications of the results for practice, policy, and future research. | 20 |
| **OTHER INFORMATION** | | |  |
| Registration and protocol | 24a | Provide registration information for the review, including register name and registration number, or state that the review was not registered. | 7 |
|  | 24b | Indicate where the review protocol can be accessed, or state that a protocol was not prepared. | 7 |
|  | 24c | Describe and explain any amendments to information provided at registration or in the protocol. | 7 |
| Support | 25 | Describe sources of financial or non-financial support for the review, and the role of the funders or sponsors in the review. | 21 |
| Competing interests | 26 | Declare any competing interests of review authors. | 21 |
| Availability of data, code and other materials | 27 | Report which of the following are publicly available and where they can be found: template data collection forms; data extracted from included studies; data used for all analyses; analytic code; any other materials used in the review. | 21 |

The current checklist followed the latest PRISMA 2020 guideline.(1)

**Supplemental Table 2: Keyword used in each database and search result**

| Database | Keyword | Filter | Date | Result |
| --- | --- | --- | --- | --- |
| PubMed | (heart failure) AND (omega 3 OR omega3 OR omega‐3 fatty acids OR omega‐3 fatty acid OR omega3 fatty acids OR omega3 fatty acid OR docosahexaenoic OR dha OR eicosapentaenoic OR epa OR polyunsaturated OR polyunsaturated fatty acids OR polyunsaturated fatty acid OR LCPUFA* OR PUFA* OR ω-3 OR ω3 OR n3 fatty acid OR n3 fatty acids OR n-3 fatty acid OR n-3 fatty acids OR omega‐6 fatty acids OR omega‐6 fatty acid OR omega6 fatty acids OR omega6 fatty acid) AND (random OR randomized OR randomised) | N/A | 2024/9/13 | 392 |
| ClinicalKey | (heart failure) AND (omega‐3 fatty acid OR docosahexaenoic OR eicosapentaenoic) AND (random OR randomized OR randomised) | N/A | 2024/9/13 | 696 |
| Cochrane CENTRAL | (heart failure) AND (omega 3 OR omega3 OR omega‐3 fatty acids OR omega‐3 fatty acid OR omega3 fatty acids OR omega3 fatty acid OR docosahexaenoic OR dha OR eicosapentaenoic OR epa OR polyunsaturated OR polyunsaturated fatty acids OR polyunsaturated fatty acid OR LCPUFA* OR PUFA* OR ω-3 OR ω3 OR n3 fatty acid OR n3 fatty acids OR n-3 fatty acid OR n-3 fatty acids OR omega‐6 fatty acids OR omega‐6 fatty acid OR omega6 fatty acids OR omega6 fatty acid) AND (random OR randomized OR randomised) | N/A | 2024/9/13 | 213 |
| Embase | (heart failure) AND (omega‐3 fatty acid OR docosahexaenoic OR eicosapentaenoic) AND (random OR randomized OR randomised) | N/A | 2024/9/13 | 576 |
| ProQuest | (heart failure) AND (omega‐3 fatty acid OR docosahexaenoic OR eicosapentaenoic) AND (random OR randomized OR randomised) | N/A | 2024/9/13 | 6425 |
| ScienceDirect | (heart failure) AND (omega‐3 fatty acid OR docosahexaenoic OR eicosapentaenoic) AND (random OR randomized OR randomised) | research article | 2024/9/13 | 2508 |
| Web of Science | (heart failure) AND (omega‐3 fatty acid OR docosahexaenoic OR eicosapentaenoic) AND (random OR randomized OR randomised) | N/A | 2024/9/13 | 177 |
| ClinicalTrials.gov | (heart failure) AND (omega‐3 fatty acid OR docosahexaenoic OR eicosapentaenoic) AND (random OR randomized OR randomised) | N/A | 2024/9/13 | 1 |

Abbreviation: N/A: not applied

**Supplemental Table 3: Excluded studies and reason**

| Reason | Numbers | References |
| --- | --- | --- |
| Animal study | 1 | (2) |
| Duplicate sample source with other included study | 4 | (3-6) |
| Exclude patients with heart failure | 3 | (7-9) |
| Inadequate randomization with significantly different severity of heart failure in baseline between experimental group and control group | 1 | (10) |
| Meta-analysis | 8 | (11-18) |
| Not provide information of specific EPA/DHA ratio | 1 | (19) |
| Not randomized controlled trial | 17 | (20-36) |
| Not randomized to polyunsaturated fatty acid supplementation | 6 | (37-42) |
| Not related to target outcome | 4 | (43-46) |
| Not specific to patients with heart failure | 9 | (47-55) |
| Study protocol but not result of a trial | 2 | (56, 57) |

**Supplemental Table 4A: League table of the subgroup of changes of LVEF in short-term treatment duration**

| High_n3PUFA_EPA_dominant | . | . | . | 0.76 [-0.04; 1.57] | . | . |
| --- | --- | --- | --- | --- | --- | --- |
| 0.24 [-1.15; 1.63] | Medium_n3PUFA_DHA_dominant | . | . | 0.53 [-0.61; 1.66] | . | . |
| 0.34 [-1.12; 1.80] | 0.10 [-1.56; 1.76] | Extreme_High_n3PUFA_EPA_dominant | 0.28 [-0.94; 1.51] | 0.43 [-0.79; 1.64] | . | . |
| 0.62 [-0.83; 2.07] | 0.38 [-1.27; 2.04] | 0.28 [-0.94; 1.51] | Medium_n3PUFA_EPA_dominant | 0.14 [-1.06; 1.34] | . | . |
| 0.76 [-0.04; 1.57] | 0.53 [-0.61; 1.66] | 0.43 [-0.79; 1.64] | 0.14 [-1.06; 1.34] | Control | 0.19 [-1.06; 1.44] | 1.00 [-0.26; 2.26] |
| 0.95 [-0.54; 2.45] | 0.72 [-0.98; 2.41] | 0.62 [-1.13; 2.36] | 0.33 [-1.40; 2.07] | 0.19 [-1.06; 1.44] | High_n3PUFA_DHA_dominant | . |
| ***1.76 [ 0.26; 3.26]** | 1.53 [-0.17; 3.23] | 1.43 [-0.33; 3.18] | 1.14 [-0.60; 2.89] | 1.00 [-0.26; 2.26] | 0.81 [-0.97; 2.59] | Extreme_High_Equal |

Data present as SMD [95%CIs]. Pairwise (upper-right portion) and network (lower-left portion) meta-analysis results are presented as estimate effect sizes for the outcome of changes of LVEF in patients with heart failure. Interventions are reported in order of mean ranking of beneficial effect on improvement of LVEF, and outcomes are expressed as standardized mean difference (SMD) (95% confidence intervals) (95%CIs). For the pairwise meta-analyses, SMD of more than 0 indicate that the treatment specified in the row got more beneficial effect than that specified in the column. For the network meta-analysis (NMA), SMD of more than 0 indicate that the treatment specified in the column got more beneficial effect than that specified in the row. Bold results marked with * indicate statistical significance.

**Supplemental Table 4B: League table of the subgroup of changes of LVEF in long-term treatment duration**

| High_n3PUFA_DHA_dominant | . | . | ***2.12 [ 1.70; 2.55]** |
| --- | --- | --- | --- |
| ***1.46 [ 0.77; 2.14]** | High_n3PUFA_EPA_dominant | . | ***0.67 [ 0.13; 1.21]** |
| ***2.06 [ 1.61; 2.52]** | ***0.60 [ 0.04; 1.17]** | Medium_n3PUFA_EPA_dominant | 0.06 [-0.10; 0.22] |
| ***2.12 [ 1.70; 2.55]** | ***0.67 [ 0.13; 1.21]** | 0.06 [-0.10; 0.22] | Control |

Data present as SMD [95%CIs]. Pairwise (upper-right portion) and network (lower-left portion) meta-analysis results are presented as estimate effect sizes for the outcome of changes of LVEF in patients with heart failure. Interventions are reported in order of mean ranking of beneficial effect on changes of LVEF, and outcomes are expressed as standardized mean difference (SMD) (95% confidence intervals) (95%CIs). For the pairwise meta-analyses, SMD of more than 0 indicate that the treatment specified in the row got more beneficial effect than that specified in the column. For the network meta-analysis (NMA), SMD of more than 0 indicate that the treatment specified in the column got more beneficial effect than that specified in the row. Bold results marked with * indicate statistical significance.

**Supplemental Table 4C: League table of the secondary outcome: changes of changes of peak VO_2_**

| Medium_n3PUFA_DHA_dominant | . | . | . | ***1.10 [ 0.47; 1.74]** | . | . |
| --- | --- | --- | --- | --- | --- | --- |
| 0.26 [-0.72; 1.25] | Extreme_High_n3PUFA_EPA_dominant | . | 0.62 [-0.15; 1.39] | ***0.84 [ 0.09; 1.59]** | . | . |
| 0.33 [-0.40; 1.05] | 0.06 [-0.77; 0.90] | High_n3PUFA_DHA_dominant | . | ***0.78 [ 0.42; 1.13]** | . | . |
| 0.88 [-0.07; 1.84] | 0.62 [-0.15; 1.39] | 0.56 [-0.24; 1.36] | Medium_n3PUFA_EPA_dominant | 0.22 [-0.50; 0.94] | . | . |
| ***1.10 [ 0.47; 1.74]** | ***0.84 [ 0.09; 1.59]** | ***0.78 [ 0.42; 1.13]** | 0.22 [-0.50; 0.94] | Control | 0.10 [-0.43; 0.62] | 0.50 [-0.29; 1.28] |
| ***1.20 [ 0.37; 2.02]** | ***0.94 [ 0.02; 1.85]** | ***0.87 [ 0.24; 1.50]** | 0.31 [-0.58; 1.21] | 0.10 [-0.43; 0.62] | High_n3PUFA_EPA_dominant | . |
| ***1.60 [ 0.59; 2.61]** | ***1.34 [ 0.25; 2.42]** | ***1.27 [ 0.41; 2.13]** | 0.72 [-0.35; 1.78] | 0.50 [-0.29; 1.28] | 0.40 [-0.54; 1.34] | Extreme_High_Equal |

Data present as SMD [95%CIs]. Pairwise (upper-right portion) and network (lower-left portion) meta-analysis results are presented as estimate effect sizes for the outcome of changes of peak VO_2_ in patients with heart failure. Interventions are reported in order of mean ranking of beneficial effect on changes of peak VO_2_, and outcomes are expressed as standardized mean difference (SMD) (95% confidence intervals) (95%CIs). For the pairwise meta-analyses, SMD of more than 0 indicate that the treatment specified in the row got more beneficial effect than that specified in the column. For the network meta-analysis (NMA), SMD of more than 0 indicate that the treatment specified in the column got more beneficial effect than that specified in the row. Bold results marked with * indicate statistical significance.

**Supplemental Table 4D: League table of the secondary outcome: changes of blood BNP levels**

| High_n3PUFA_EPA_dominant | . | -0.81 [-2.21; 0.59] | . |
| --- | --- | --- | --- |
| -0.83 [-3.19; 1.53] | Medium_n3PUFA_EPA_dominant | 0.22 [-1.81; 2.24] | -0.31 [-2.36; 1.73] |
| -0.81 [-2.21; 0.59] | 0.02 [-1.88; 1.91] | Control | -0.12 [-1.54; 1.29] |
| -0.94 [-2.93; 1.05] | -0.11 [-2.01; 1.80] | -0.12 [-1.54; 1.29] | Extreme_High_n3PUFA_EPA_dominant |

Data present as SMD [95%CIs]. Pairwise (upper-right portion) and network (lower-left portion) meta-analysis results are presented as estimate effect sizes for the outcome of changes of blood BNP levels in patients with heart failure. Interventions are reported in order of mean ranking of beneficial effect on changes of blood BNP levels, and outcomes are expressed as standardized mean difference (SMD) (95% confidence intervals) (95%CIs). For the pairwise meta-analyses, SMD of less than 0 indicate that the treatment specified in the row got more beneficial effect than that specified in the column. For the network meta-analysis (NMA), SMD of less than 0 indicate that the treatment specified in the column got more beneficial effect than that specified in the row. Bold results marked with * indicate statistical significance.

**Supplemental Table 4E: League table of the secondary outcome: changes of quality of life**

| Extreme_High_Equal | . | . | 0.71 [-0.08; 1.50] |
| --- | --- | --- | --- |
| 0.50 [-0.46; 1.46] | High_n3PUFA_EPA_dominant | 0.15 [-0.41; 0.71] | 0.21 [-0.33; 0.75] |
| 0.65 [-0.31; 1.62] | 0.15 [-0.41; 0.71] | High_pure_EPA | 0.06 [-0.49; 0.60] |
| 0.71 [-0.08; 1.50] | 0.21 [-0.33; 0.75] | 0.06 [-0.49; 0.60] | Control |

Data present as OR [95%CIs]. Pairwise (upper-right portion) and network (lower-left portion) meta-analysis results are presented as estimate effect sizes for the outcome of changes of quality of life in patients with heart failure. Interventions are reported in order of mean ranking of beneficial effect on changes of quality of life, and outcomes are expressed as standardized mean difference (SMD) (95% confidence intervals) (95%CIs). For the pairwise meta-analyses, SMD of more than zero indicate that the treatment specified in the row got more beneficial effect than that specified in the column. For the network meta-analysis (NMA), SMD of more than zero indicate that the treatment specified in the column got more beneficial effect than that specified in the row. Bold results marked with * indicate statistical significance.

**Supplemental Table 4F: League table of the safety profile: drop-out rate**

| Medium_n3PUFA_DHA_dominant | . | . | 0.48 [0.03; 9.07] | . | . | . |
| --- | --- | --- | --- | --- | --- | --- |
| 0.67 [0.03; 16.06] | High_n3PUFA_EPA_dominant | . | 0.71 [0.22; 2.29] | . | . | 0.57 [0.08; 3.85] |
| 0.58 [0.02; 15.23] | 0.86 [0.14; 5.41] | Medium_n3PUFA_EPA_dominant | 0.72 [0.16; 3.16] | 1.68 [0.16; 17.43] | . | . |
| 0.48 [0.03; 9.07] | 0.71 [0.22; 2.29] | 0.82 [0.20; 3.36] | Control | 0.29 [0.03; 2.76] | 0.78 [0.06; 9.65] | 0.57 [0.08; 3.85] |
| 0.40 [0.01; 13.35] | 0.59 [0.06; 5.58] | 0.68 [0.10; 4.63] | 0.83 [0.12; 5.67] | Extreme_High_n3PUFA_EPA_dominant | . | . |
| 0.37 [0.01; 17.86] | 0.55 [0.03; 8.86] | 0.64 [0.04; 11.43] | 0.78 [0.06; 9.65] | 0.93 [0.04; 22.08] | Extreme_High_Equal | . |
| 0.32 [0.01; 9.93] | 0.48 [0.08; 2.75] | 0.56 [0.06; 5.25] | 0.68 [0.12; 3.89] | 0.81 [0.06; 10.87] | 0.87 [0.04; 18.69] | High_pure_EPA |

Data present as OR [95%CIs]. Pairwise (upper-right portion) and network (lower-left portion) meta-analysis results are presented as estimate effect sizes for the outcome of drop-out rate in patients with heart failure. Interventions are reported in order of mean ranking of preferred safety profile, and outcomes are expressed as odds ratio (OR) (95% confidence intervals) (95%CIs). For the pairwise meta-analyses, OR of less than 1 indicate that the treatment specified in the row got more preferred safety profile than that specified in the column. For the network meta-analysis (NMA), OR of less than 1 indicate that the treatment specified in the column got more preferred safety profile than that specified in the row. Bold results marked with * indicate statistical significance.

**Supplemental Table 4G: League table of the safety profile: all-cause mortality**

| High_pure_EPA | . | . | 0.32 [0.01; 8.23] | 0.32 [0.01; 8.23] | . |
| --- | --- | --- | --- | --- | --- |
| 0.37 [0.01; 9.48] | Extreme_High_pure_EPA | . | . | 0.88 [0.69; 1.12] | . |
| 0.35 [0.01; 9.01] | 0.96 [0.74; 1.25] | Medium_n3PUFA_EPA_dominant | . | 0.92 [0.82; 1.02] | . |
| 0.32 [0.01; 8.23] | 0.88 [0.09; 8.94] | 0.92 [0.09; 9.25] | High_n3PUFA_EPA_dominant | 1.00 [0.10; 10.08] | . |
| 0.32 [0.01; 8.23] | 0.88 [0.69; 1.12] | 0.92 [0.82; 1.02] | 1.00 [0.10; 10.08] | Control | 0.43 [0.04; 5.16] |
| 0.14 [0.00; 8.27] | 0.38 [0.03; 4.58] | 0.40 [0.03; 4.73] | 0.43 [0.01; 12.84] | 0.43 [0.04; 5.16] | Extreme_High_n3PUFA_EPA_dominant |

Data present as OR [95%CIs]. Pairwise (upper-right portion) and network (lower-left portion) meta-analysis results are presented as estimate effect sizes for the outcome of all-cause mortality rate in patients with heart failure. Interventions are reported in order of mean ranking of preferred safety profile, and outcomes are expressed as odds ratio (OR) (95% confidence intervals) (95%CIs). For the pairwise meta-analyses, OR of less than 1 indicate that the treatment specified in the row got more preferred safety profile than that specified in the column. For the network meta-analysis (NMA), OR of less than 1 indicate that the treatment specified in the column got more preferred safety profile than that specified in the row. Bold results marked with * indicate statistical significance.

*Abbreviation: 95%CIs: 95% confidence intervals; BNP: brain natriuretic peptide; DHA: docosahexaenoic acid; EPA: eicosapentaenoic acid; Extreme_High_Equal: extreme high dosage (at least 4000 mg/day) n3PUFA treatment with composition of EPA/DHA ratio equal to 1; Extreme_High_n3PUFA_EPA_dominant: extreme high dosage (at least 4000 mg/day) n3PUFA treatment with composition of EPA predominant; Extreme_High_pure_EPA: extreme high dosage (at least 4000 mg/day) n3PUFA treatment with composition of pure EPA; High_n3PUFA_DHA_dominant: high dosage (at least 2000 mg/day but less than 4000 mg/day) n3PUFA treatment with composition of DHA predominant; High_n3PUFA_EPA_dominant: high dosage (at least 2000 mg/day but less than 4000 mg/day) n3PUFA treatment with composition of EPA predominant; High_pure_EPA: high dosage (at least 2000 mg/day but less than 4000 mg/day) n3PUFA treatment with composition of pure EPA; LVEF: left ventricular ejection fraction; Medium_n3PUFA_DHA_dominant: medium dosage (at least 1000 mg/day but less than 2000 mg/day) n3PUFA treatment with composition of DHA predominant; Medium_n3PUFA_EPA_dominant: medium dosage (at least 1000 mg/day but less than 2000 mg/day) n3PUFA treatment with composition of EPA predominant; n3PUFA: omega-3 polyunsaturated fatty acid; NMA: network meta-analysis; OR: odds ratio; peak VO2: peak oxygen consumption; RCT: randomized controlled trial; SMD: standardized mean difference*

**Supplemental Table 5: inconsistency within the network meta-analysis of primary outcome: changes of left ventricular ejection fraction**

| Comparison | No.Studies | NMA | Direct | Indirect | Difference | Diff_95CI_lower | Diff_95CI_upper | P Value |
| --- | --- | --- | --- | --- | --- | --- | --- | --- |
| Extreme_High_Equal:Control | 1 | -1 | -1 | NA | NA | NA | NA | NA |
| Extreme_High_n3PUFA_EPA_dominant:Control | 1 | 0.404611 | 0.426941 | 0.260902 | 0.166039 | -4.0262 | 4.35828 | 0.938125 |
| High_n3PUFA_DHA_dominant:Control | 2 | 1.086167 | 1.086167 | NA | NA | NA | NA | NA |
| High_n3PUFA_EPA_dominant:Control | 4 | 0.729313 | 0.729313 | NA | NA | NA | NA | NA |
| Medium_n3PUFA_DHA_dominant:Control | 1 | 0.525588 | 0.525588 | NA | NA | NA | NA | NA |
| Medium_n3PUFA_EPA_dominant:Control | 2 | 0.097078 | 0.097078 | NA | NA | NA | NA | NA |
| Extreme_High_Equal:Extreme_High_n3PUFA_EPA_dominant | 0 | -1.40461 | NA | -1.40461 | NA | NA | NA | NA |
| Extreme_High_Equal:High_n3PUFA_DHA_dominant | 0 | -2.08617 | NA | -2.08617 | NA | NA | NA | NA |
| Extreme_High_Equal:High_n3PUFA_EPA_dominant | 0 | -1.72931 | NA | -1.72931 | NA | NA | NA | NA |
| Extreme_High_Equal:Medium_n3PUFA_DHA_dominant | 0 | -1.52559 | NA | -1.52559 | NA | NA | NA | NA |
| Extreme_High_Equal:Medium_n3PUFA_EPA_dominant | 0 | -1.09708 | NA | -1.09708 | NA | NA | NA | NA |
| Extreme_High_n3PUFA_EPA_dominant:High_n3PUFA_DHA_dominant | 0 | -0.68156 | NA | -0.68156 | NA | NA | NA | NA |
| Extreme_High_n3PUFA_EPA_dominant:High_n3PUFA_EPA_dominant | 0 | -0.3247 | NA | -0.3247 | NA | NA | NA | NA |
| Extreme_High_n3PUFA_EPA_dominant:Medium_n3PUFA_DHA_dominant | 0 | -0.12098 | NA | -0.12098 | NA | NA | NA | NA |
| Extreme_High_n3PUFA_EPA_dominant:Medium_n3PUFA_EPA_dominant | 1 | 0.307534 | 0.284627 | 0.448524 | -0.1639 | -4.30206 | 3.974265 | 0.938125 |
| High_n3PUFA_DHA_dominant:High_n3PUFA_EPA_dominant | 0 | 0.356854 | NA | 0.356854 | NA | NA | NA | NA |
| High_n3PUFA_DHA_dominant:Medium_n3PUFA_DHA_dominant | 0 | 0.560579 | NA | 0.560579 | NA | NA | NA | NA |
| High_n3PUFA_DHA_dominant:Medium_n3PUFA_EPA_dominant | 0 | 0.989089 | NA | 0.989089 | NA | NA | NA | NA |
| High_n3PUFA_EPA_dominant:Medium_n3PUFA_DHA_dominant | 0 | 0.203725 | NA | 0.203725 | NA | NA | NA | NA |
| High_n3PUFA_EPA_dominant:Medium_n3PUFA_EPA_dominant | 0 | 0.632236 | NA | 0.632236 | NA | NA | NA | NA |
| Medium_n3PUFA_DHA_dominant:Medium_n3PUFA_EPA_dominant | 0 | 0.428511 | NA | 0.428511 | NA | NA | NA | NA |

*Abbreviation:* *95%CIs: 95% confidence intervals; DHA: docosahexaenoic acid; EPA: eicosapentaenoic acid; Extreme_High_Equal: extreme high dosage (at least 4000 mg/day) n3PUFA treatment with composition of EPA/DHA ratio equal to 1; Extreme_High_n3PUFA_EPA_dominant: extreme high dosage (at least 4000 mg/day) n3PUFA treatment with composition of EPA predominant; Extreme_High_pure_EPA: extreme high dosage (at least 4000 mg/day) n3PUFA treatment with composition of pure EPA; High_n3PUFA_DHA_dominant: high dosage (at least 2000 mg/day but less than 4000 mg/day) n3PUFA treatment with composition of DHA predominant; High_n3PUFA_EPA_dominant: high dosage (at least 2000 mg/day but less than 4000 mg/day) n3PUFA treatment with composition of EPA predominant; High_pure_EPA: high dosage (at least 2000 mg/day but less than 4000 mg/day) n3PUFA treatment with composition of pure EPA; Medium_n3PUFA_DHA_dominant: medium dosage (at least 1000 mg/day but less than 2000 mg/day) n3PUFA treatment with composition of DHA predominant; Medium_n3PUFA_EPA_dominant: medium dosage (at least 1000 mg/day but less than 2000 mg/day) n3PUFA treatment with composition of EPA predominant; n3PUFA: omega-3 polyunsaturated fatty acid; NMA: network meta-analysis; OR: odds ratio; RCT: randomized controlled trial; SMD: standardized mean difference*

**Reference list of supplement materials:**

1. Page MJ, McKenzie JE, Bossuyt PM, Boutron I, Hoffmann TC, Mulrow CD et al. The PRISMA 2020 statement: an updated guideline for reporting systematic reviews. Bmj 2021;372:n71.

2. Eclov JA, Qian Q, Redetzke R, Chen Q, Wu SC, Healy CL et al. EPA, not DHA, prevents fibrosis in pressure overload-induced heart failure: potential role of free fatty acid receptor 4. Journal of lipid research 2015;56(12):2297-2308.

3. Aleksova A, Masson S, Maggioni AP, Lucci D, Fabbri G, Beretta L et al. n-3 polyunsaturated fatty acids and atrial fibrillation in patients with chronic heart failure: the GISSI-HF trial. Eur J Heart Fail 2013;15(11):1289-1295.

4. La Rovere MT, Staszewsky L, Barlera S, Maestri R, Mezzani A, Midi P et al. n-3PUFA and Holter-derived autonomic variables in patients with heart failure: data from the Gruppo Italiano per lo Studio della Sopravvivenza nell'Insufficienza Cardiaca (GISSI-HF) Holter substudy. Heart Rhythm 2013;10(2):226-232.

5. Finzi AA, Latini R, Barlera S, Rossi MG, Ruggeri A, Mezzani A et al. Effects of n-3 polyunsaturated fatty acids on malignant ventricular arrhythmias in patients with chronic heart failure and implantable cardioverter-defibrillators: A substudy of the Gruppo Italiano per lo Studio della Sopravvivenza nell'Insufficienza Cardiaca (GISSI-HF) trial. Am Heart J 2011;161(2):338-343 e331.

6. Ghio S, Scelsi L, Latini R, Masson S, Eleuteri E, Palvarini M et al. Effects of n-3 polyunsaturated fatty acids and of rosuvastatin on left ventricular function in chronic heart failure: a substudy of GISSI-HF trial. Eur J Heart Fail 2010;12(12):1345-1353.

7. Djousse L, Cook NR, Kim E, Walter J, Al-Ramady OT, Luttmann-Gibson H et al. Diabetes Mellitus, Race, and Effects of Omega-3 Fatty Acids on Incidence of Heart Failure Hospitalization. JACC Heart Fail 2022;10(4):227-234.

8. Djousse L, Cook NR, Kim E, Bodar V, Walter J, Bubes V et al. Supplementation With Vitamin D and Omega-3 Fatty Acids and Incidence of Heart Failure Hospitalization: VITAL-Heart Failure. Circulation 2020;141(9):784-786.

9. Manson JE, Cook NR, Lee IM, Christen W, Bassuk SS, Mora S et al. Vitamin D Supplements and Prevention of Cancer and Cardiovascular Disease. N Engl J Med 2019;380(1):33-44.

10. Chrysohoou C, Metallinos G, Georgiopoulos G, Mendrinos D, Papanikolaou A, Magkas N et al. Short term omega-3 polyunsaturated fatty acid supplementation induces favorable changes in right ventricle function and diastolic filling pressure in patients with chronic heart failure; A randomized clinical trial. Vascul Pharmacol 2016;79:43-50.

11. Seth J, Sharma S, Leong CJ, Rabkin SW. Eicosapentaenoic Acid (EPA) and Docosahexaenoic Acid (DHA) Ameliorate Heart Failure through Reductions in Oxidative Stress: A Systematic Review and Meta-Analysis. Antioxidants (Basel) 2024;13(8).

12. Dwiputra B, Ambari AM, Desandri DR, Purwowiyoto BS, Radi B, Pandhita BAW et al. The Effect of Omega-3 Supplementation on Heart Failure Outcome: A Meta-Analysis of Randomized Clinical Trial. J Lipid Atheroscler 2024;13(2):89-96.

13. Prokopidis K, Therdyothin A, Giannos P, Morwani-Mangnani J, Ferentinos P, Mitropoulos A et al. Does omega-3 supplementation improve the inflammatory profile of patients with heart failure? a systematic review and meta-analysis. Heart Fail Rev 2023;28(6):1417-1425.

14. Zheng S, Qiu M, Wu JHY, Pan XF, Liu X, Sun L et al. Long-chain omega-3 polyunsaturated fatty acids and the risk of heart failure. Ther Adv Chronic Dis 2022;13:20406223221081616.

15. Liu J, Meng Q, Zheng L, Yu P, Hu H, Zhuang R et al. Effect of n-3 PUFA on left ventricular remodelling in chronic heart failure: a systematic review and meta-analysis. Br J Nutr 2022:1-10.

16. Barbarawi M, Lakshman H, Barbarawi O, Alabdouh A, Al Kasasbeh M, Djousse L et al. Omega-3 supplementation and heart failure: A meta-analysis of 12 trials including 81,364 participants. Contemporary clinical trials 2021;107:106458.

17. Wang C, Xiong B, Huang J. The Role of Omega-3 Polyunsaturated Fatty Acids in Heart Failure: A Meta-Analysis of Randomised Controlled Trials. Nutrients 2016;9(1).

18. Djousse L, Akinkuolie AO, Wu JH, Ding EL, Gaziano JM. Fish consumption, omega-3 fatty acids and risk of heart failure: a meta-analysis. Clin Nutr 2012;31(6):846-853.

19. Kojuri J, Ostovan MA, Rezaian GR, Archin Dialameh P, Zamiri N, Sharifkazemi MB et al. Effect of omega-3 on brain natriuretic peptide and echocardiographic findings in heart failure: Double-blind placebo-controlled randomized trial. J Cardiovasc Dis Res 2013;4(1):20-24.

20. Akesson A, Donat-Vargas C, Berglund M, Glynn A, Wolk A, Kippler M. Dietary exposure to polychlorinated biphenyls and risk of heart failure - A population-based prospective cohort study. Environ Int 2019;126:1-6.

21. Carbone S, Canada JM, Buckley LF, Trankle CR, Billingsley HE, Dixon DL et al. Dietary Fat, Sugar Consumption, and Cardiorespiratory Fitness in Patients With Heart Failure With Preserved Ejection Fraction. JACC Basic Transl Sci 2017;2(5):513-525.

22. Kohashi K, Nakagomi A, Saiki Y, Morisawa T, Kosugi M, Kusama Y et al. Effects of eicosapentaenoic acid on the levels of inflammatory markers, cardiac function and long-term prognosis in chronic heart failure patients with dyslipidemia. J Atheroscler Thromb 2014;21(7):712-729.

23. Mozaffarian D, Lemaitre RN, King IB, Song X, Spiegelman D, Sacks FM et al. Circulating long-chain omega-3 fatty acids and incidence of congestive heart failure in older adults: the cardiovascular health study: a cohort study. Ann Intern Med 2011;155(3):160-170.

24. Belin RJ, Greenland P, Martin L, Oberman A, Tinker L, Robinson J et al. Fish intake and the risk of incident heart failure: the Women's Health Initiative. Circ Heart Fail 2011;4(4):404-413.

25. Cowie MR, Cure S, Bianic F, McGuire A, Goodall G, Tavazzi L. Cost-effectiveness of highly purified omega-3 polyunsaturated fatty acid ethyl esters in the treatment of chronic heart failure: results of Markov modelling in a UK setting. Eur J Heart Fail 2011;13(6):681-689.

26. Levitan EB, Wolk A, Mittleman MA. Fatty fish, marine omega-3 fatty acids and incidence of heart failure. Eur J Clin Nutr 2010;64(6):587-594.

27. Levitan EB, Wolk A, Mittleman MA. Fish consumption, marine omega-3 fatty acids, and incidence of heart failure: a population-based prospective study of middle-aged and elderly men. Eur Heart J 2009;30(12):1495-1500.

28. Yamagishi K, Iso H, Date C, Fukui M, Wakai K, Kikuchi S et al. Fish, omega-3 polyunsaturated fatty acids, and mortality from cardiovascular diseases in a nationwide community-based cohort of Japanese men and women the JACC (Japan Collaborative Cohort Study for Evaluation of Cancer Risk) Study. J Am Coll Cardiol 2008;52(12):988-996.

29. Mozaffarian D, Bryson CL, Lemaitre RN, Burke GL, Siscovick DS. Fish intake and risk of incident heart failure. J Am Coll Cardiol 2005;45(12):2015-2021.

30. Le VT, Knight S, Watrous JD, Najhawan M, Dao K, McCubrey RO et al. Higher docosahexaenoic acid levels lower the protective impact of eicosapentaenoic acid on long-term major cardiovascular events. Front Cardiovasc Med 2023;10:1229130.

31. Matsuo N, Miyoshi T, Takaishi A, Kishinoue T, Yasuhara K, Tanimoto M et al. High Plasma Docosahexaenoic Acid Associated to Better Prognoses of Patients with Acute Decompensated Heart Failure with Preserved Ejection Fraction. Nutrients 2021;13(2).

32. Block RC, Liu L, Herrington DM, Huang S, Tsai MY, O'Connell TD et al. Predicting Risk for Incident Heart Failure With Omega-3 Fatty Acids: From MESA. JACC Heart Fail 2019;7(8):651-661.

33. Ouchi S, Miyazaki T, Shimada K, Sugita Y, Shimizu M, Murata A et al. Low Docosahexaenoic Acid, Dihomo-Gamma-Linolenic Acid, and Arachidonic Acid Levels Associated with Long-Term Mortality in Patients with Acute Decompensated Heart Failure in Different Nutritional Statuses. Nutrients 2017;9(9).

34. Hara M, Sakata Y, Nakatani D, Suna S, Usami M, Matsumoto S et al. Low levels of serum n-3 polyunsaturated fatty acids are associated with worse heart failure-free survival in patients after acute myocardial infarction. Circ J 2013;77(1):153-162.

35. Dijkstra SC, Brouwer IA, van Rooij FJ, Hofman A, Witteman JC, Geleijnse JM. Intake of very long chain n-3 fatty acids from fish and the incidence of heart failure: the Rotterdam Study. Eur J Heart Fail 2009;11(10):922-928.

36. Yamagishi K, Nettleton JA, Folsom AR, Investigators AS. Plasma fatty acid composition and incident heart failure in middle-aged adults: the Atherosclerosis Risk in Communities (ARIC) Study. Am Heart J 2008;156(5):965-974.

37. Lechner K, Scherr J, Lorenz E, Lechner B, Haller B, Krannich A et al. Omega-3 fatty acid blood levels are inversely associated with cardiometabolic risk factors in HFpEF patients: the Aldo-DHF randomized controlled trial. Clin Res Cardiol 2022;111(3):308-321.

38. Colin-Ramirez E, Castillo-Martinez L, Orea-Tejeda A, Zheng Y, Westerhout CM, Ezekowitz JA. Dietary fatty acids intake and mortality in patients with heart failure. Nutrition 2014;30(11-12):1366-1371.

39. Strand E, Pedersen ER, Svingen GF, Schartum-Hansen H, Rebnord EW, Bjorndal B et al. Dietary intake of n-3 long-chain polyunsaturated fatty acids and risk of myocardial infarction in coronary artery disease patients with or without diabetes mellitus: a prospective cohort study. BMC Med 2013;11:216.

40. Petrone AB, Weir N, Hanson NQ, Glynn R, Tsai MY, Gaziano JM et al. Omega-6 fatty acids and risk of heart failure in the Physicians' Health Study. Am J Clin Nutr 2013;97(1):66-71.

41. Wilk JB, Tsai MY, Hanson NQ, Gaziano JM, Djousse L. Plasma and dietary omega-3 fatty acids, fish intake, and heart failure risk in the Physicians' Health Study. Am J Clin Nutr 2012;96(4):882-888.

42. Jiang W, Oken H, Fiuzat M, Shaw LK, Martsberger C, Kuchibhatla M et al. Plasma omega-3 polyunsaturated fatty acids and survival in patients with chronic heart failure and major depressive disorder. J Cardiovasc Transl Res 2012;5(1):92-99.

43. Moertl D, Berger R, Hammer A, Hutuleac R, Koppensteiner R, Kopp CW et al. Dose-dependent decrease of platelet activation and tissue factor by omega-3 polyunsaturated fatty acids in patients with advanced chronic heart failure. Thrombosis and haemostasis 2011;106(3):457-465.

44. Eschen O, Christensen JH, MT LAR, Romano P, Sala P, Schmidt EB. Effects of marine n-3 fatty acids on circulating levels of soluble adhesion molecules in patients with chronic heart failure. Cell Mol Biol (Noisy-le-grand) 2010;56(1):45-51.

45. Mehra MR, Lavie CJ, Ventura HO, Milani RV. Fish oils produce anti-inflammatory effects and improve body weight in severe heart failure. J Heart Lung Transplant 2006;25(7):834-838.

46. Morgan DR, Dixon LJ, Hanratty CG, El-Sherbeeny N, Hamilton PB, McGrath LT et al. Effects of dietary omega-3 fatty acid supplementation on endothelium-dependent vasodilation in patients with chronic heart failure. Am J Cardiol 2006;97(4):547-551.

47. Campos-Staffico AM, Costa APR, Carvalho LSF, Moura FA, Santos SN, Coelho-Filho OR et al. Omega-3 intake is associated with attenuated inflammatory response and cardiac remodeling after myocardial infarction. Nutr J 2019;18(1):29.

48. Bhatt DL, Steg PG, Miller M, Brinton EA, Jacobson TA, Ketchum SB et al. Cardiovascular Risk Reduction with Icosapent Ethyl for Hypertriglyceridemia. N Engl J Med 2019;380(1):11-22.

49. Nosaka K, Miyoshi T, Iwamoto M, Kajiya M, Okawa K, Tsukuda S et al. Early initiation of eicosapentaenoic acid and statin treatment is associated with better clinical outcomes than statin alone in patients with acute coronary syndromes: 1-year outcomes of a randomized controlled study. Int J Cardiol 2017;228:173-179.

50. Heydari B, Abdullah S, Pottala JV, Shah R, Abbasi S, Mandry D et al. Effect of Omega-3 Acid Ethyl Esters on Left Ventricular Remodeling After Acute Myocardial Infarction: The OMEGA-REMODEL Randomized Clinical Trial. Circulation 2016;134(5):378-391.

51. Risk, Prevention Study Collaborative G, Roncaglioni MC, Tombesi M, Avanzini F, Barlera S et al. n-3 fatty acids in patients with multiple cardiovascular risk factors. N Engl J Med 2013;368(19):1800-1808.

52. Investigators OT, Bosch J, Gerstein HC, Dagenais GR, Diaz R, Dyal L et al. n-3 fatty acids and cardiovascular outcomes in patients with dysglycemia. N Engl J Med 2012;367(4):309-318.

53. Brouwer IA, Zock PL, Camm AJ, Bocker D, Hauer RN, Wever EF et al. Effect of fish oil on ventricular tachyarrhythmia and death in patients with implantable cardioverter defibrillators: the Study on Omega-3 Fatty Acids and Ventricular Arrhythmia (SOFA) randomized trial. Jama 2006;295(22):2613-2619.

54. Skou HA, Toft E, Christensen JH, Hansen JB, Dyerberg J, Schmidt EB. N-3 fatty acids and cardiac function after myocardial infarction in Denmark. International journal of circumpolar health 2001;60(3):360-365.

55. Sacks FM, Stone PH, Gibson CM, Silverman DI, Rosner B, Pasternak RC. Controlled trial of fish oil for regression of human coronary atherosclerosis. HARP Research Group. J Am Coll Cardiol 1995;25(7):1492-1498.

56. Lennie TA, Moser DK, Biddle MJ, Welsh D, Bruckner GG, Thomas DT et al. Nutrition intervention to decrease symptoms in patients with advanced heart failure. Res Nurs Health 2013;36(2):120-145.

57. Manson JE, Bassuk SS, Lee IM, Cook NR, Albert MA, Gordon D et al. The VITamin D and OmegA-3 TriaL (VITAL): rationale and design of a large randomized controlled trial of vitamin D and marine omega-3 fatty acid supplements for the primary prevention of cancer and cardiovascular disease. Contemporary clinical trials 2012;33(1):159-171.
